# Molecular cylinders with donor–acceptor structure and swinging motion†

**DOI:** 10.1039/d4sc05849f

**Published:** 2024-10-23

**Authors:** Ke Li, Satoshi Yoshida, Ryo Yakushiji, Xingchi Liu, Chang Ge, Zhuofan Xu, Yong Ni, Xiaonan Ma, Jishan Wu, Sota Sato, Zhe Sun

**Affiliations:** a Institute of Molecular Plus, Department of Chemistry, Tianjin University and Haihe Laboratory of Sustainable Chemical Transformations 92 Weijin Road Tianjin 300072 China zhesun@tju.edu.cn; b Integrated Molecular Structure Analysis Laboratory, Department of Applied Chemistry, School of Engineering, The University of Tokyo 6-6-2 Kashiwanoha, Kashiwa-shi Chiba 277-0882 Japan satosota@appchem.t.u-tokyo.ac.jp; c Institute for Molecular Science (IMS) 5-1 Higashiyama, Myodaiji Okazaki Aichi 444-8787 Japan; d Department of Chemistry, Southern University of Science and Technology Shenzhen Guangdong 518055 China; e Department of Chemistry, National University of Singapore 3 Science Drive 3 117543 Singapore

## Abstract

The construction of three-dimensional nanocarbon structures with well-defined molecular dynamics is a challenging yet rewarding task in material science and supramolecular chemistry. Herein, we report the synthesis of two highly defective, nitrogen-doped molecular cylinders, namely MC1 and MC2, with a length of 1.4 nm and 2.7 nm, respectively. These molecular cylinders are constructed by connecting the cycloparaphenylene endcaps and fused aromatic pillars using a cyclocondensation reaction, affording a distinct donor–acceptor structure. An X-ray crystallographic analysis reveals a tilted cylindrical shape for MC1, and nuclear magnetic resonance spectroscopy and calculations indicate the occurrence of a dynamic swinging motion in solution. The elongation of conjugation in the cylinders attenuates the charge transfer character in the first excited state, resulting in remarkable length-dependent photophysical properties.

## Abstract 2

Two nanometer-sized, nitrogen-containing molecular cylinders were synthesized by connecting the cycloparaphenylene and fused aromatic moieties using cyclocondensation reaction.

## Graphical abstract

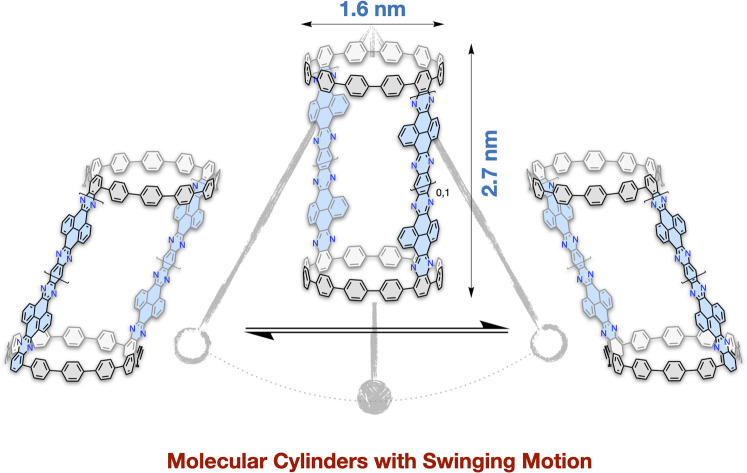

## Introduction

Complex three-dimensional (3D) nanocarbon structures with dynamic motions are intriguing yet challenging synthetic targets.^1–3^ In particular, controlling molecular motions has led to molecular machines as a flourishing research field,^4–9^ whereas the restriction of molecular motions often produce structurally unique 3D organic architectures, such as cages,^10,11^ belts,^12–15^ Möbius strips,^16–19^ and interlocked systems.^20–23^ The creation of distinctive molecular topologies is often associated with the emergence of chirality,^24–26^ unique optoelectronic properties,^27^ and encapsulation capabilities,^28,29^ which stimulates the development of synthetic chemistry,^30,31^ material science,^32^ and supramolecular chemistry.^33^ Among the 3D organic motifs, molecular cylinders are particularly attractive, and their construction represents a formidable task. Using phenine and porphyrin as building blocks, Isobe^34^ and Anderson^35^ prepared nanometer size phenine cylinders and porphyrin cylinders, respectively, using meticulously designed synthetic methods. However, the rational synthesis, structural elucidation, and investigation of molecular cylinders with dynamic motions remain challenging.

**Fig. 1:**
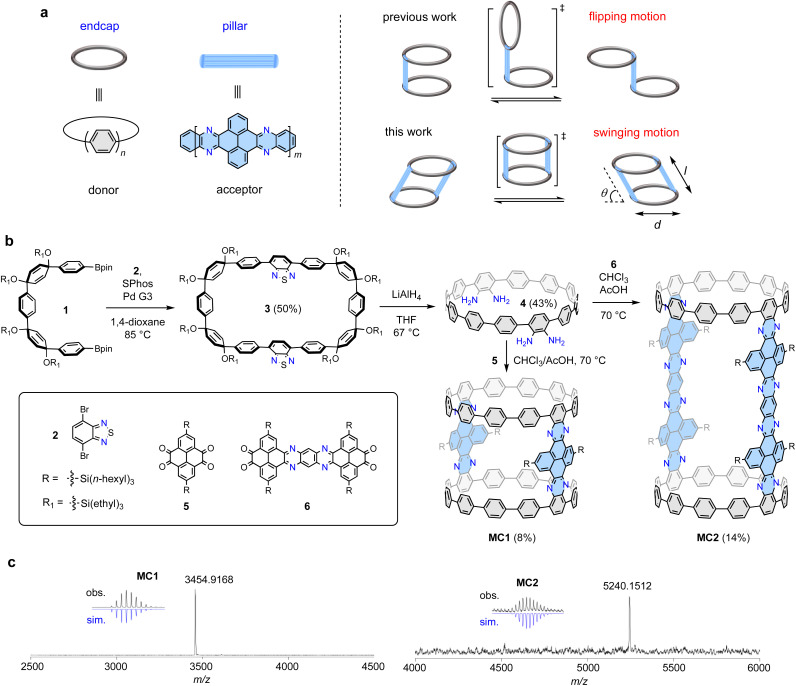
Synthesis and structure of molecular cylinders MC1 and MC2. (a) Molecular design and dynamic motion. (b) Synthesis of MC1 and MC2. (c) High-resolution MALDI-TOF MS spectra.

Recently, our group have developed a modular cyclocondensation approach for the π-lengthening of the cycloparaphenylenes (CPPs), which also allows for the incorporation of N atoms to produce donor–acceptor (D–A) structures with bright redshifted emission.^36^ With CPP as an endcap and a N-doped aromatic moiety (NAM) as a pillar, a bridged dimeric CPP exhibiting distinctive flipping motion by virtue of the free rotating single bonds connecting the CPP and the NAM units was synthesized (Fig. 1a).^37^ Following this design concept, we envisioned that the installation of an additional pillar at the opposite site would stop the flipping motion and force a persistent cylindrical shape. However, under this circumstance, the rotation of the single bonds connecting the CPP and the NAM units might not be fully restricted, resulting in a swinging motion. The molecular cylinders can be described by diameter (*d*), length (*l*), and angular displacement (*θ*) from the vertical equilibrium position (transition state shown in Fig. 1a), which is a phenomenon rarely observed in 3D nanocarbons.

Herein, we present the synthesis, structural elucidation, and photophysical properties of two discrete molecular cylinders, MC1 and MC2, which possess tunable length, swinging dynamics, and length-dependent photophysical properties. These cylindrical molecules can be geometrically viewed as molecular fragments of N-doped (12,12)-carbon nanotubes (CNTs),^38^ but they swing away from the perfect cylindrical shape in the crystalline state. The incorporation of both electron-donating CPP endcaps and electron-withdrawing NAM pillars into the molecular backbone leads to unique photophysical properties.^39^

## Results and discussion

### Design and synthesis

The construction of the proposed cylindrical structure hinges on a cyclocondensation reaction between a CPP tetraamine compound and an aromatic tetraketone compound. As shown in Fig. 1b, the synthesis of the key tetraamine intermediate started from a Suzuki macrocyclization reaction between the previously reported U-shaped compound 1 and dibromobenzothiadiazole 2. The reaction concentration was carefully controlled at 1 mM to ensure an optimal reaction yield of 50% for macrocycle 3. Subsequently, tetraamine intermediate 4 was obtained in 43% yield using a strategy previously developed by us, which involved a one-pot reductive aromatization–sulfur extrusion strategy using LiAlH_4_.^37^ A cyclocondensation reaction between 4 and tetraketone 5 afforded a mixture of cyclic and linear products, from which the desired product MC1 was isolated in 8% yield using gel-permeable chromatography (GPC). Compound 5 was synthesized according to a reported method,^40^ and a branched trihexylsilyl substituent was used to ensure a sufficient solubility for the target molecules. To maintain a highly diluted reaction environment that ensures macrocyclization is favored over undesired polymerization, 4 and 5 were simultaneously injected to a stirring solvent over a period of 2 h (see ESI† for details). Elongated tetraketone 6 was then prepared according to a reported method,^41^ and the longer cylinder MC2 was synthesized and isolated in 14% yield under the same reaction conditions and operational technique. The slightly higher yield of MC2 than MC1 may be due to its better solubility provided by branched trihexylsilyl substituent. The reaction sequence comprises only three linear steps from available starting materials; therefore, this concise synthetic approach can be expected to be readily applicable to other tetraketones to produce a variety of cylinders. The molecular structures of MC1 and MC2 were unambiguously confirmed by means of X-ray crystallography and spectroscopy analyses (*vide infra*). The chemical compositions of MC1 (C_248_H_252_N_8_Si_4_) and MC2 (C_364_H_420_N_16_Si_8_) were confirmed *via* high-resolution matrixassisted laser desorption/ionization (MALDI) mass spectroscopy, which showed a mass/charge ratio (*m*/*z*) of 3454.9168 and 5240.1512, respectively, matching well with the theoretical values of 3454.9115 and 5240.1584 (Fig. 1c).

### X-ray crystallographic analysis

**Fig. 2:**
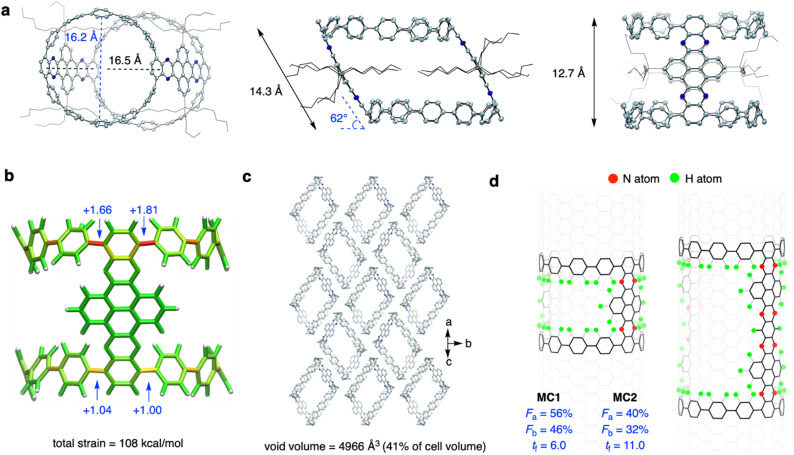
Crystal structure of MC1 and structural analysis. (a) Single crystal structures from top and side views. (b) StrainVis analysis of MC1 without substituents from DFT optimization. (c) Packing structure of MC1. Aliphatic substituents and solvent molecules are omitted for clarity. (d) Mapping of MC1 and MC2 on CNT and the representative vector-based descriptions.

Yellow prism crystals of MC1 and red ellipsoidal crystals of MC2 were obtained *via* slow diffusion of *n*-hexane into a CHCl_3_ solution at −20 °C (Fig. S1†). However, the fragibility of the MC2 crystals prevented us from performing X-ray crystallographic measurements, and the MC1 crystal gave weak diffraction patterns on a conventional X-ray diffractometer. Fortunately, measurements could be performed on a monochromated X-ray beam at KEK PF BL-17A beamline, affording data with sufficient resolution. As shown in Fig. 2a, the crystal structure of MC1 showed a tilted cylindrical shape (a parallelogram shape from the side view) of nanometer size with an averaged diameter (*d*) of 16.4 Å, a tilting angle (*θ*) of 62°, a length (*l*) of 14.3 Å, and a height (*h*) of 12.7 Å. Such a geometry is beneficial for alleviating the strain of the sterically congested aliphatic chains. The endcapping CPP unit exhibited a slight deviation from the cycle, and the torsional angles of the aryl rings ranged from 23° to 51° (Fig. S2†). The decrease in symmetry from a perfect cylinder to a tilted cylinder also reduced the volume from 2308 Å^3^ (*θ* = 90°) to 2031 Å^3^ (*θ* = 62°) (Fig. S3†). Measurement of the π-orbital axis vectors (POAV)^42^ indicated that the ipso-carbons have a structural deformation from planarity, with POAV values ranging from 2.79° to 3.88° (Fig. 2a and S4†). These values are larger than those of [12]CPP (averaged at 2.7°),^43^ suggesting a more deformed CPP structure when confined into the tilted cylinder. The POAV values are slightly larger for the upper site (with acute angle of the parallelogram) than for the bottom site (with obtuse angle of the parallelogram), which is in agreement with the strain energies obtained by performing a StrainVis analysis (Fig. 2b),^44^ and can be attributed to the intrusion of the NAM pillar into the cavity of the cylinder. The total strain energy calculated from the crystal structure was 108 kcal mol^−1^, which is much lower than the strain energy of 281 kcal mol^−1^ for the perfect cylinder (Fig. S5†).

The MC1 molecules were packed in the *P*2_1_/*c* space group (Table S1†), in which the cylinders assembled in a top-to-side fashion with the electron-rich CPP endcaps located spatially close to the electron-withdrawing NAM pillar (Fig. 2c and S6†). This packing mode is different from that of Isobe's cylinders^34^ with a layered stacking of molecules, which could be ascribed to the unique D–A structure of MC1. The intercylinder interactions are mainly CH–π and π–π interactions between the donor and acceptor parts, and the aliphatic chains of the adjacent cylinders and the hexane solvent molecules fill up the cavity of the central molecule (Fig. S6†). The analysis of the interior and interstitial void space resulted in a void volume of 4966 Å^3^, which occupies 41% of the cell volume (Fig. 2c). Owing to its porous nature, the MC1 crystal could find application in guest adsorption and catalysis.^45^

Then, we analyzed the defective nature and N-doping of MC1 and MC2 using the geometric measures for finite nanotube molecules developed by Isobe *et al.*^46^ Theoretically, the perfect upright cylinders can be mapped into the (12,12)-CNT structure, and the vector indices of length index (*t*_f_), atom-filling index (*F*_a_), and bond-filling index (*F*_b_) can be applied. The *t*_f_ values measures the length of the cylinder, and the *F*_a_ and *F*_b_ values quantify the occupancy of atoms and bonds of the cylinder in the mother CNT structure, which determines the defectiveness. As shown in Fig. 2d, the *F*_a_ values of MC1 and MC2 are 56% and 40% and *F*_b_ values of MC1 and MC2 are 46% and 32%, respectively, which are much smaller than those of the most defective finite nanotube molecule reported to date (*F*_a_ = 67% and *F*_b_ = 57%),^34^ revealing that MC1 and MC2 possess a highly hollow structure. The *t*_f_ values of MC1 and MC2 are 6.0 and 11.0, respectively, which correspond to an actual length of 14.9 Å and 27.4 Å by multiplying the lattice constant, in consistent with the value obtained from the crystal structure. Note that the value of 11.0 for MC2 exceeds that of the longest finite nanotube molecule reported (*t*_f_ = 7.0). Taken together, these results indicate that the molecular cylinder MC2 is the longest, most defective finite nanotube molecule synthesized so far. In addition, using an oblique coordinate system,^47^ the positions of 8 nitrogen atoms in MC1 and 16 nitrogen atoms in MC2 can be pinpointed (Fig. S7†).

### Solution-phase structure and dynamics

**Fig. 3:**
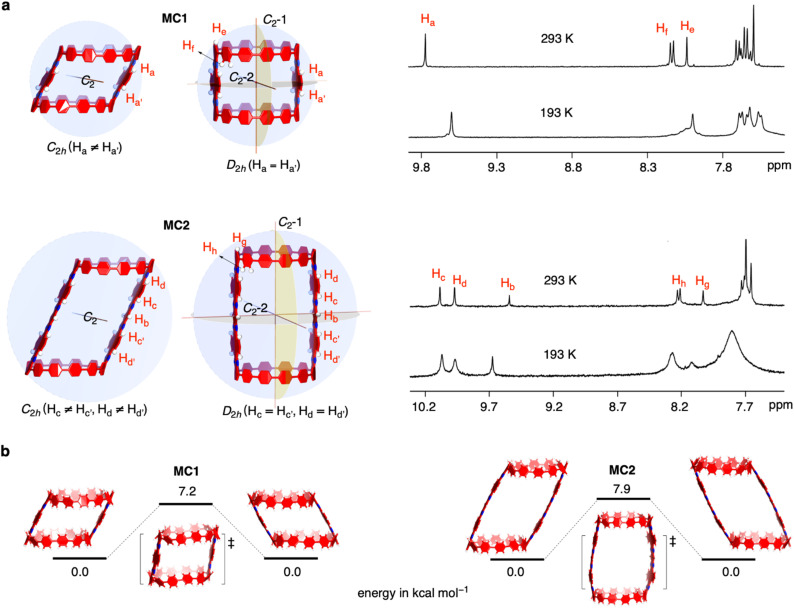
NMR spectra and DFT calculations. (a) Variable temperature NMR spectra of MC1 and MC2 measured in dichloromethane-d_2_/CS_2_ and MC2 measured in tetrahydrofuran-d_8_, respectively. Illustrative structures with different symmetries are shown. (b) Calculated stationary and transition state structures for MC1 and MC2.

The solution-phase structures of MC1 and MC2 were determined using nuclear magnetic resonance (NMR) spectroscopy. The introduction of solubilizing alkyl chains allowed obtaining well-resolved ^1^H NMR signals at 293 K. A simple, symmetric spectral pattern was observed for both MC1 and MC2, in which the protons at the upper (H_a_, H_c_, H_d_) are equivalent with those at the bottom 
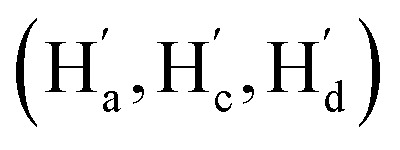
, inconsistent with a *C*_2h_ symmetric tilted cylinder structure observed in the crystalline state (Fig. 3a). This observation suggests that the *C*_2h_ tilted cylinder structure is not persistent in the solution phase; instead, the molecule swing back and forth like a pendulum on the NMR time scale to give a time-averaged *D*_2h_ structure. Lowering the temperature led to broadening of the signals, implying a slowdown of the dynamic motion (Fig. 3a and S8, S9†). Unfortunately, the H_a_/H_b_/H_c_ and 
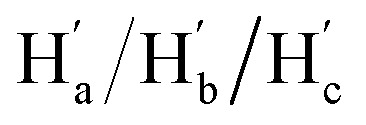
 protons of the *C*_2h_ structure could not be distinguished even at the instrumental temperature limit of 193 K. The proton signals on the NAM pillar could be assigned using two-dimensional COSY and NOESY spectra (Fig. S10 and S11†). In general, the protons from the NAM panel appeared at the relatively lower field than those from the CPP units due to a considerable deshielding from the fused aromatic moiety.

Next, we investigated the swinging dynamics *via* theoretical calculations. First, we performed a torsional scan analysis on the energetics by freezing the dihedral angles between CPP and NAM at the semiempirical PM6 level to locate the transition state (TS, Fig. S12†),^48^ and more precise energetics were obtained by performing geometry optimizations with density functional theory (DFT) calculations at the M062X/6-31G(d,p) level (Fig. 3b). TS structures with nearly upright geometries were found with an activation energy of 7.2 kcal mol^−1^ for MC1 and 7.9 kcal mol^−1^ for MC2, respectively. These values are lower than the activation energy for the ring-flipping of the single-pillar CPP dimer (10.1 kcal mol^−1^), which is consistent with a coalescence temperature close or below 193 K, as observed in variable-temperature NMR measurements.

### Photophysical properties

**Fig. 4:**
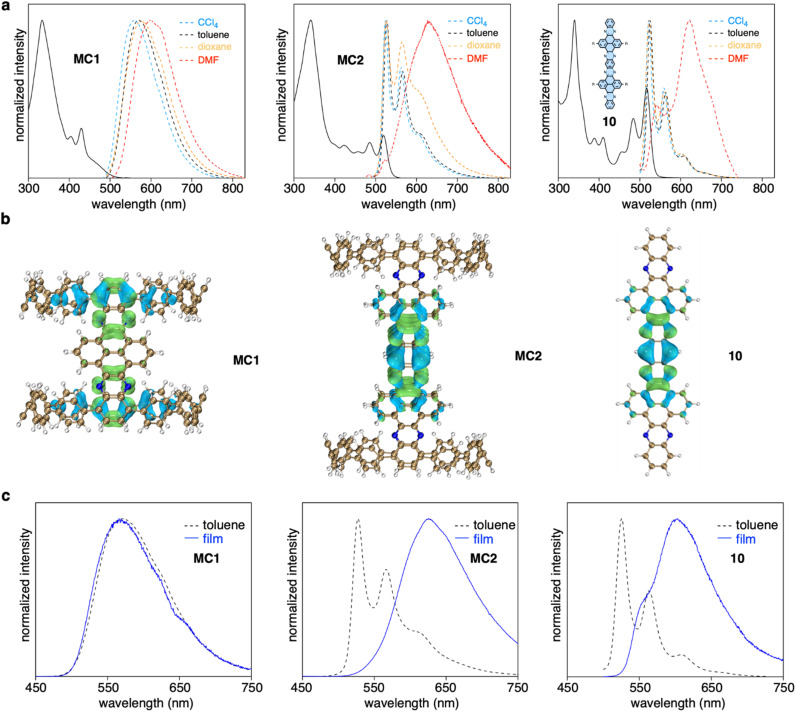
Photophysical properties. (a) UV-vis absorption (solid line) in toluene and fluorescence (dashed line) spectra of MC1, MC2, and 10. (b) Electron–hole analysis of the S_0_ → S_1_ excitation. Blue and green regions denote hole and electron distributions, respectively (isovalue = 0.001). (c) Fluorescence spectra measured in toluene solution and in the film state.

MC1 and MC2 exhibited distinct length-dependent photophysical properties. The absorption spectra of MC1 and MC2 displayed a prominent peak at around 340 nm, with shoulder peaks extending to 500 nm for MC1 and 550 nm for MC2 (Fig. 4a). However, time-dependent DFT calculations suggested that the seemingly similar absorption spectra possessed different origins. For MC1, the maximum absorption (*λ*_max_) at 333 nm corresponds to the electronic transitions from HOMO−1 to LUMO+8/LUMO+9, and the shoulder peaks at lower energy regime stem from the partially allowed HOMO to LUMO transition (Fig. S13 and Table S2†). The occupied and unoccupied molecular orbitals involved in the transitions are spatially separated and localized at the CPP and NAM units, respectively, similar to our previously reported D–A nanohoops.^36^ In contrast, the *λ*_max_ and shoulder peaks of MC2 are due to HOMO−6 to LUMO+7/HOMO−7 to LUMO+6 and HOMO−8 to LUMO/HOMO−9 to LUMO+1 transitions, with the orbitals being exclusively distributed on the NAM unit (Fig. S14 and Table S3†). This result suggests that the NAM moiety serves as a chromophore for MC2, whereas the contribution from CPP is negligible. The absorption spectrum of substructure 10, which was synthesized for comparison, was very similar to that of MC2, confirming that NAM is responsible for the observed absorption of MC2.

The fluorescence spectra of MC1 and MC2 were also distinct. A clear bathochromic shift from 560 to 600 nm was observed for MC1 when the fluorescence was measured in solvents with increased polarity (Fig. 4a). The *E*_T_ (30) plot gave a good linearity, which suggests a positive solvatofluorochromism, *i.e.* the dipole moment of S_1_ is higher than S_0_ state (Fig. S15†).^49^ Meanwhile, MC2 showed typical LE fluorescence spectra with vibronic progression in CCl_4_, toluene, and dioxane, whiledisplayed a redshifted spectrum in *N*,*N*-dimethylformamide (DMF). This behavior is also reminiscent of the solvent dependence of 10. A quantitative electron–hole analysis^50^ on the critical S_0_ → S_1_ excitation was performed to gain further insight into the observed emission (Fig. 4b, S16, and Table S4†). Both CPP and NAM units of MC1 were found to participate in S_1_ (CT) excitation, whereas the S_1_ excitation was confined on the central NAM moiety for MC2 and 10. As a result, the S_1_ state of MC1 possessed 41% of charge transfer (CT) contribution and 59% of local excitation (LE) contribution, whereas only LE contributed to the S_1_ state of MC2 and 10. This explains the existence of solvatofluorochromism of MC1 and the spectral featuring of vibronic progression for MC2.^51^ The anomalous spectra in DMF can be explained in terms of the aggregation caused by intermolecular interactions in a polar solvent.^52^ This hypothesis was further supported by measuring the fluorescence in toluene solution and in the film state (Fig. 4c). In both cases, the spectral pattern was almost identical for MC1, but a redshifted behavior from solution to the film state was observed for MC2 and 10. The fluorescence quantum yield (*Φ*) of MC1 was determined to be 63% in toluene (Fig. S17†), which is surprisingly high for a nanometer-sized molecular cylinder. This result, together with the unique D–A structure and length dependence, suggests that such molecular cylinders are good candidates for the exploration of new photophysical mechanisms and applications.^53^

## Conclusions

In summary, two N-doped molecular cylinders, MC1 and MC2, with a well-defined D–A structure and tunable length were synthesized using cyclocondensation as a key macrocyclization approach. Their structures can be viewed as molecular fragments of N-doped (12,12)-CNTs. An X-ray crystallographic analysis revealed the tilted cylindrical geometry and porous nature of the crystal packing, and a swinging motion was revealed in solution by spectroscopic and computational methods. The molecular cylinders exhibited unique length-dependent photophysical properties. Moreover, the shorter cylinder possessed considerable CT characteristics, and the elongation of the aromatic pillar weakens the contribution from the CPP units. The molecules reported here may serve as discrete molecular models for the understanding of the effect of heteroatom doping in CNTs,^54,55^ and open a new avenue for the development of cylindrical shaped molecular hosts, dynamic systems, and functional materials.^56^

## Author contributions

K. L. and Z. S. conceived the design. Z. S., S. S., and J. W. supervised the project. K. L., X. L., and C. G. performed the synthesis, compound characterization and data analysis. S. Y., R. Y., and S. S. performed the crystallographic studies. All authors analyzed and discussed the results, and K. L. and Z. S. wrote the manuscript.

## Conflicts of interest

There are no conflicts to declare.

## Supplementary Material

SC-015-D4SC05849F-s001

SC-015-D4SC05849F-s002

## Data Availability

The data supporting this article have been included as part of the ESI.†

## References

[cit1] Feng L., Astumian R. D., Stoddart J. F. (2022). Nat. Rev. Chem.

[cit2] Wang X., Jia F., Yang L.-P., Zhou H., Jiang W. (2020). Chem. Soc. Rev..

[cit3] Segawa Y., Ito H., Itami K. (2016). Nat. Rev. Mater..

[cit4] Bissell R. A., Córdova E., Kaifer A. E., Stoddart J. F. (1994). Nature.

[cit5] Feng L., Qiu Y., Guo Q.-H., Chen Z., Seale J. S. W., He K., Wu H., Feng Y., Farha O. K., Astumian R. D., Stoddart J. F. (2021). Science.

[cit6] Zhang L., Qiu Y., Liu W.-G., Chen H., Shen D., Song B., Cai K., Wu H., Jiao Y., Feng Y., Seale J. S. W., Pezzato C., Tian J., Tan Y., Chen X.-Y., Guo Q.-H., Stern C. L., Philp D., Astumian III R. D., Goddard W. A., Stoddart J. F. (2023). Nature.

[cit7] Huck N. P. M., Jager W. F., de Lange B., Feringa B. L. (1996). Science.

[cit8] Koumura N., Zijlstra R. W. J., van Delden R. A., Harada N., Feringa B. L. (1999). Nature.

[cit9] Štacko P., Kistemaker J. C. M., van Leeuwen T., Chang M.-C., Otten E., Feringa B. L. (2017). Science.

[cit10] Sun Q.-F., Iwasa J., Ogawa D., Ishido Y., Sato S., Ozeki T., Sei Y., Yamaguchi K., Fujita M. (2010). Science.

[cit11] Ni Y., Gopalakrishna T. Y., Phan H., Kim T., Herng T. S., Han Y., Tao T., Ding J., Kim D., Wu J. (2020). Nat. Chem..

[cit12] Povie G., Segawa Y., Nishihara T., Miyauchi Y., Itami K. (2017). Science.

[cit13] Cheung K. Y., Watanabe K., Segawa Y., Itami K. (2021). Nat. Chem..

[cit14] Han Y., Dong S., Shao J., Fan W., Chi C. (2021). Angew. Chem., Int. Ed..

[cit15] Cheung K. Y., Gui S., Deng C., Liang H., Xia Z., Liu Z., Chi L., Miao Q. (2019). Chem.

[cit16] Fan W., Fukunaga T. M., Wu S., Han Y., Zhou Q., Wang J., Li Z., Hou X., Wei H., Ni Y., Isobe H., Wu J. (2023). Nat. Synth..

[cit17] Segawa Y., Watanabe T., Yamanoue K., Kuwayama M., Watanabe K., Pirillo J., Hijikata Y., Itami K. (2022). Nat. Synth..

[cit18] Ajami D., Oeckler O., Simon A., Herges R. (2003). Nature.

[cit19] Tanaka Y., Saito S., Mori S., Aratani N., Shinokubo H., Shibata N., Higuchi Y., Yoon Z. S., Kim K. S., Noh S. B., Park J. K., Kim D., Osuka A. (2008). Angew. Chem., Int. Ed..

[cit20] Segawa Y., Kuwayama M., Hijikata Y., Fushimi M., Nishihara T., Pirillo J., Shirasaki J., Kubota N., Itami K. (2019). Science.

[cit21] May J. H., Van Raden J. M., Maust R. L., Zakharov L. N., Jasti R. (2023). Nat. Chem..

[cit22] Fan Y.-Y., Chen D., Huang Z.-A., Zhu J., Tung C.-H., Wu L.-Z., Cong H. (2018). Nat. Commun..

[cit23] Bu A., Zhao Y., Xiao H., Tung C.-H., Wu L.-Z., Cong H. (2022). Angew. Chem., Int. Ed..

[cit24] Hitosugi S., Nakanishi W., Yamasaki T., Isobe H. (2011). Nat. Commun..

[cit25] Tian Y., Guo Y., Dong X., Wan X., Cheng K.-H., Chang R., Li S., Cao X., Chan Y.-T., Sue A. C.-H. (2023). Nat. Synth..

[cit26] Sato S., Yoshii A., Takahashi S., Furumi S., Takeuchi M., Isobe H. (2017). Proc. Natl. Acad. Sci. U. S. A..

[cit27] Zhang X., Liu H., Zhuang G., Yang S., Du P. (2022). Nat. Commun..

[cit28] Iwamoto T., Watanabe Y., Sadahiro T., Haino T., Yamago S. (2011). Angew. Chem., Int. Ed..

[cit29] Ubasart E., Borodin O., Fuertes-Espinosa C., Xu Y., García-Simón C., Gómez L., Juanhuix J., Gándara F., Imaz I., Maspoch D., von Delius M., Ribas X. (2021). Nat. Chem..

[cit30] Hitosugi S., Matsumoto A., Kaimori Y., Iizuka R., Soai K., Isobe H. (2014). Org. Lett..

[cit31] Kayahara E., Hayashi T., Takeuchi K., Ozawa F., Ashida K., Ogoshi S., Yamago S. (2018). Angew. Chem., Int. Ed..

[cit32] Leonhardt E. J., Jasti R. (2019). Nat. Rev. Chem.

[cit33] Xu Y., von Delius M. (2020). Angew. Chem., Int. Ed..

[cit34] Sun Z., Ikemoto K., Fukunaga T. M., Koretsune T., Arita R., Sato S., Isobe H. (2019). Science.

[cit35] Hoffmann M., Wilson C. J., Odell B., Anderson H. L. (2007). Angew. Chem., Int. Ed..

[cit36] Deng H., Guo Z., Wang Y., Li K., Zhou Q., Ge C., Xu Z., Sato S., Ma X., Sun Z. (2022). Chem. Sci..

[cit37] Li K., Xu Z., Deng H., Zhou Z., Dang Y., Sun Z. (2021). Angew. Chem., Int. Ed..

[cit38] Ikemoto K., Yang S., Naito H., Kotani M., Sato S., Isobe H. (2020). Nat. Commun..

[cit39] Hermann M., Wassy D., Esser B. (2021). Angew. Chem., Int. Ed..

[cit40] More S., Bhosale R., Choudhary S., Mateo-Alonso A. (2012). Org. Lett..

[cit41] Mateo-Alonso A., Kulisic N., Valenti G., Marcaccio M., Paolucci F., Prato M. (2010). Chem.–Asian J..

[cit42] Haddon R. C. (1986). Pure Appl. Chem..

[cit43] Segawa Y., Miyamoto S., Omachi H., Matsuura S., Šenel P., Sasamori T., Tokitoh N., Itami K. (2011). Angew. Chem., Int. Ed..

[cit44] Colwell C. E., Price T. W., Stauch T., Jasti R. (2020). Chem. Sci..

[cit45] Sun M.-H., Huang S.-Z., Chen L.-H., Li Y., Yang X.-Y., Yuan Z.-Y., Su B.-L. (2016). Chem. Soc. Rev..

[cit46] Matsuno T., Naito H., Hitosugi S., Sato S., Kotani M., Isobe H. (2014). Pure Appl. Chem..

[cit47] Saito R. , Dresselhaus G. and Dresselhaus M. S., Physical properties of carbon nanotubes, Imperial College Press, London, 1998

[cit48] Xia J., Golder M. R., Foster M. E., Wong B. M., Jasti R. (2012). J. Am. Chem. Soc..

[cit49] Reichardt C. (1994). Chem. Rev..

[cit50] Liu Z., Lu T., Chen Q. (2020). Carbon.

[cit51] Li W., Liu D., Shen F., Ma D., Wang Z., Feng T., Xu Y., Yang B., Ma Y. (2012). Adv. Funct. Mater..

[cit52] Wasserfallen D., Kastler M., Pisula W., Hofer W. A., Fogel Y., Wang Z., Müllen K. (2006). J. Am. Chem. Soc..

[cit53] Uoyama H., Goushi K., Shizu K., Nomura H., Adachi C. (2012). Nature.

[cit54] Ayala P., Arenal R., Rümmeli M., Rubio A., Pichler T. (2010). Carbon.

[cit55] Inagaki M., Toyoda M., Soneda Y., Morishita T. (2018). Carbon.

[cit56] Bunz U. H. F. (2015). Acc. Chem. Res..

