# Supplementary material for: Molecular cylinders with donor–acceptor structure and swinging motion
Source: Chem Sci. 2024 Oct 23;15(45):18832–9. doi: 10.1039/d4sc05849f (PMC11506531; doi:10.1039/d4sc05849f)
Supplement: SC-015-D4SC05849F-s001 [file SC-015-D4SC05849F-s001.pdf]

Supplementary Information (SI) for Chemical Science.
This journal is © The Royal Society of Chemistry 2024

Supplementary Information

Molecular cylinders with donor-acceptor structure and swinging motion

Ke Li,[1] Satoshi Yoshida,[2] Ryo Yakushiji,[2] Xingchi Liu,[1] Chang Ge,[1] Zhuofan Xu,[1] Yong Ni,[4] Xiaonan Ma,[1] Jishan Wu,[5] Sota Sato,[2,3]* and Zhe Sun[1]*

[1]Institute of Molecular Plus, Department of Chemistry, Tianjin University and Haihe Laboratory of Sustainable Chemical Transformations, 92 Weijin Road, Tianjin, 300072 China;

[2]"Integrated Molecular Structure Analysis Laboratory", Department of Applied Chemistry School of Engineering, The University of Tokyo, 6-6-2 Kashiwanoha, Kashiwa-shi, Chiba 277-0882;

[3]Institute for Molecular Science (IMS), 5-1 Higashiyama, Myodaiji, Okazaki, Aichi 444-8787 Japan;

[4]Department of Chemistry, Southern University of Science and Technology, Shenzhen, Guangdong, 518055 P. R.China;

[5]Department of Chemistry, National University of Singapore, 3 Science Drive 3, 117543, Singapore

*e-mail: satosota@appchem.t.u-tokyo.ac.jp (S.S.); zhesun@tju.edu.cn (Z.S.)

Table of Contents

1. Experimental section 2

2. Structural analysis 8

3. Solution phase structural characterization 14

4. Photophysical properties 16

5. NMR spectra 21

6. Reference 29

1. Experimental section

1.1 General

All reagents were purchased from the commercial source and used without further purification. Compounds **1**[1], **7**[2] and SPhos Pd G3[3] were prepared according to literature. Anhydrous tetrahydrofuran (THF) was distilled with sodium. All experiments were performed under argon atmosphere. Column chromatography was performed with silica gel (200-300 mesh) or neutral alumina (200-300 mesh). Proton (^{1}H) NMR and carbon (^{13}C) NMR spectra were recorded on JNM-ECZ600R/S1 with tetramethylsilane (TMS) as the internal standard. Chemical shifts were given in ppm relative to residue protons ($CHCl_3$: δ 7.26 for ^{1}H, 77.16 for ^{13}C; CH_2Cl_2: δ 5.32 for ^{1}H; THF: δ 1.72, 3.58 for ^{1}H). The following abbreviations were used for multiplicities: s = singlet, d = doublet, t = triplet, q = quartet, m = multiplet). Matrix Assisted Laser Desorption Ionization (MALDI) mass spectra were performed on Bruker Daltonics Autoflex time-of-flight (TOF) equipment (*trans*-2-[3-(4-tert-butyl)-2-methyl-2-propenylidene]malononitrile (DCTB) was used as matrix for compound **3**, and 1,8,9-trihydroxyanthracene (THA) was used as matrix for compound **5**, **10**, **MC1** and **MC2**). High resolution Atmospheric Pressure Chemical Ionization (APCI) mass spectra were recorded on FT-ICR-MS Solarix 7T instrument. UV-Vis-NIR absorption spectra were recorded on SHIMADZU UV-2600 spectrophotometer. Fluorescence measurements were carried out on FL-1000 Spectrophotometer. The fluorescence quantum yields were determined in absolute values with integrating sphere.

1.2 Computational methods

DFT calculations were performed with the Gaussian09 program suite[4]. The geometry optimizations were performed at M062X/6-31G(d,p)[5,6,7] level of theory with single crystal structures as the input structures. Transition structures (TS) of **MC1** and **MC2** were optimized using QST3 method. All optimized structures were confirmed to be true minima by vibrational analysis with no imaginary frequency and all transition states were confirmed with one imaginary frequency. The hole-electron analysis were performed at M062X/6-31G(d,p) level of theory and soft with Multiwfn software[8] and the charge transfer weight (CT%) for the crucial excited states was quantitatively evaluated by calculating the fragments contributions.[9, 10] The strain visualization was calculated by using the method developed by Jasti *et al.* [11] For POAV

analysis, both pyramidalization angles (θ_p) and dihedral angles (ϕ_p) were obtained by using poav.py developed by Isobe et al. [12] The molecular models were visualized by using UCSF Chimera.[13]

1.3 Crystallographic methods

The single crystals of **MC1** suitable for X-ray crystallographic analysis were obtained by slow diffusion of *n*-hexane into a solution of sample in chloroform at −20 ºC in air. The diffraction analysis with a synchrotron X-ray source was conducted at 95 K at the beamline BL38B1 at the SPring-8 using a diffractometer equipped with a Dectris PILATUS3 6M PAD detector. The collected diffraction data were processed with the XDS software program[14]. The structures were solved by direct method using the SHELXT software program[15] and refined by full-matrix least-squares on F^2 using the SHELX-2018/3 program suite[16] running on the Yadokari-XG 2009 software program.[17] For more detailed information about diffraction data collection and refinement parameters, see Table S1. The crystallographic data were deposited in Cambridge Crystallographic Data Centre (CCDC 2363037). The data can be achieved free of charge from www.ccdc.cam.ac.uk/data_request/cif.

1.4 Synthesis

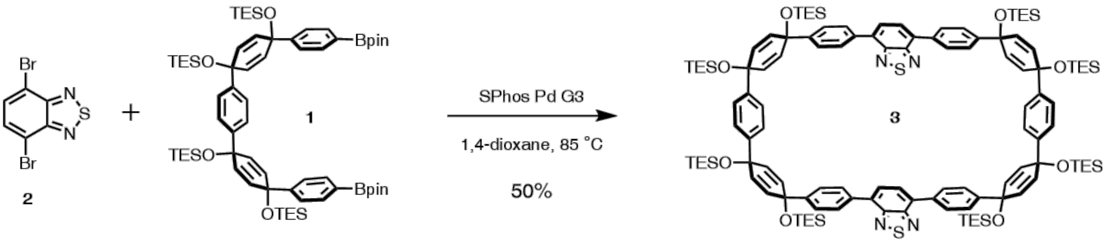

Compound **1** (394 mg, 340 μmol), **2** (100 mg, 340 μmol) and SPhos Pd G3 (27 mg, 34 μmol) were added to a 1000 mL round bottom flask equipped with a magnetic stirring bar. After the flask was evacuated and refilled with argon for three times, degassed 1,4-dioxane (340 mL) was added and the mixture was heated to 85 ºC and stirred for 10 min. Then degassed K_3PO_4 solution (34 mL, 2 M in deionized water) was added to the round bottom flask and the solution was stirred at 85 ºC for 12 h. After the reaction mixture was cooled down to room temperature, the mixture was filtered through Celite. The filtrate was dried over $MgSO_4$ and concentrated. The residue was purified by GPC (DCM as eluent) and further washed with acetone to afford

compound **3** as a yellow solid (180 mg, 50%). ^{1}H NMR (600 MHz, $CDCl_3$) δ = 7.73 (d, *J* = 8.5 Hz, 8H), 7.44 (m, 16H), 7.07 (s, 4H), 6.11 (d, *J* = 9.9 Hz, 8H), 5.98 (d, *J* = 10.0 Hz, 8H), 0.99 (t, *J* = 8.0 Hz, 36H), 0.93 (t, *J* = 7.9 Hz, 36H), 0.70 (q, *J* = 7.9 Hz, 24H), 0.57 (q, *J* = 8.0 Hz, 24H) ppm. ^{13}C NMR (150 MHz, $CDCl_3$) δ = 154.1, 146.1, 145.3, 144.9, 144.6, 136.1, 132.8, 132.7, 131.6, 131.5, 131.4, 131.1, 128.9, 128.1, 127.2, 126.0, 125.9, 125.7, 71.5, 71.2, 71.1, 71.1, 7.1, 7.0, 6.4, 6.4, 6.3 ppm. MS (MALDI-TOF) (*m/z*) $[M+H]^+$ calcd for $C_{120}H_{165}N_4O_8S_2Si_8$ 2078.0217, found 2078.1488.

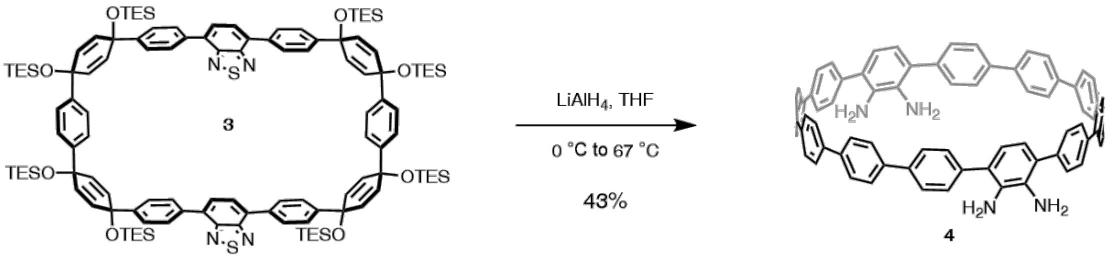

Compound **3** (300 mg, 0.144 mmol) was dissolved in dry THF (50 mL) in a 100 mL Schlenk flask under Argon atmosphere. The solution was stirred for 30 min in an ice bath. Then $LiAlH_4$ (110 mg, 2.89 mmol)) was added and the colorless solution turned to a deep purple. After stirring at 0 °C for another 30 min, the solution was transferred to an oil bath and heated to 67 °C. After 12 h, The reaction was quenched by adding deionized water dropwise, and then extracted with ethyl acetate (3 × 50 mL), washed with brine (50 mL), dried over $MgSO_4$ and concentrated. The residue was further purified by sonicating in acetone to afford compound **4** as a yellow solid (61.0 mg, 43%). ^{1}H NMR (600 MHz, $CDCl_3$) δ = 7.63 – 7.61 (m, 32H), 7.59 – 7.56 (m, 8H), 6.34 (s, 4H), 3.91 (s, 8H) ppm. DEPT 135 ^{13}C NMR (150 MHz, $CDCl_3$) δ = 128.5, 127.2, 127.1, 127.0, 123.9 ppm. HRMS (APCI-TOF) (*m/z*) $[M+H]^+$ calcd for $C_{72}H_{53}N_4$ 973.4265, found 973.4259.

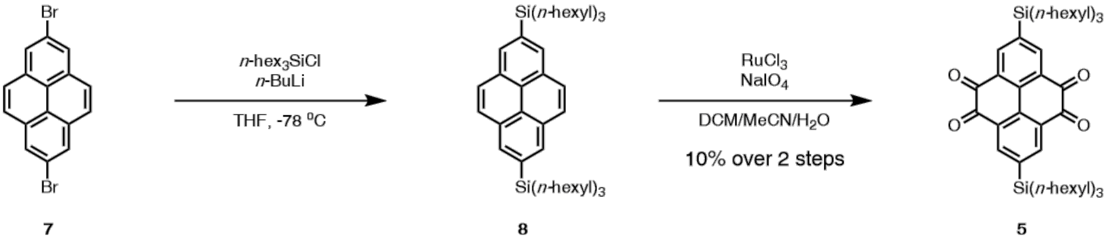

Compound **7** (1.00 g, 2.78 mmol) was dissolved in anhydrous THF (20 mL) in a 100 mL round bottom flask containing a magnetic stirring bar. After the mixture was cooled down to −78 °C,

n-butyllithium (2.89 mL, 6.94 mmol, 2.4 M in THF) was added and the mixture was stirred for 30 min. Then tri-*n*-hexylchlorosilane (2.23 mL, 8.33 mmol) was added to the reaction and stirred for 4 h. The reaction was quenched by adding deionized water dropwise, and the obtained mixture was extracted with ethyl acetate (3 × 20 mL), washed with brine (20 mL), dried over $MgSO_4$ and concentrated to crude residue containing compound **8**. The residue was then dissolved in 75 mL DCM and 75 mL CH_3CN and 25 mL deionized water in a 250 mL round bottom flask containing a magnetic stirring bar. To this mixture, $RuCl_3$ (75.1 mg, 333 μmol) and $NaIO_4$ (4.90 g, 22.9 mmol) were added and stirred for 4 h at 40 °C. The mixture was cooled down to room temperature and extracted with DCM (3 × 20 mL) followed by washed with brine (20 mL), dried over $MgSO_4$ and concentrated. The crude product was purified by column chromatography (silica gel, ethyl acetate/petroleum ether = 1/10) to afford compound **5** as an orange solid (220 mg, 10% over two steps). ^1H NMR (600 MHz, $CDCl_3$) δ = 8.54 (s, 4H), 1.37 – 1.20 (m, 48H), 0.90 – 0.84 (m, 30H) ppm. ^{13}C NMR (150 MHz, $CDCl_3$) δ = 178.6, 144.1, 142.2, 134.6, 129.7, 33.2, 31.4, 23.6, 22.5, 14.1, 11.8 ppm. MS (ESI-TOF) (*m/z*) $[M+H]^+$ calcd for $C_{52}H_{83}O_4Si_2$ 827.5824, found 827.5837. MS (MALDI-TOF) (*m/z*) $[M+H]^+$ calcd for $C_{52}H_{83}O_4Si_2$ 827.5824, found 827.7713.

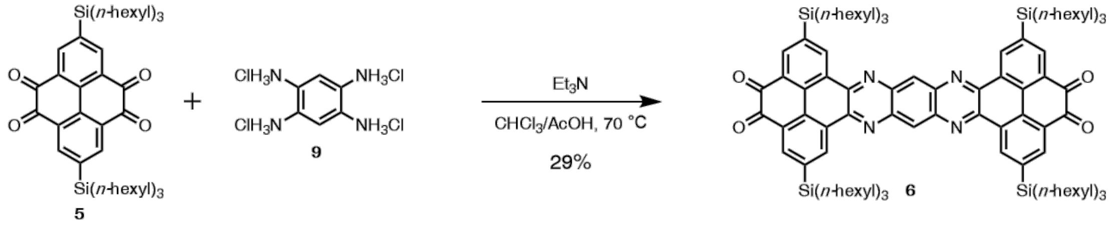

Compound **5** (87.4 mg, 0.105 mmol) and **9** (10.0 mg, 35.2 μmol) were added to a 25 mL Schlenk flask containing a magnetic stirring bar, and 4 mL degassed $CHCl_3$ and 1 mL acetic acid were injected to the flask and heated to 70 °C. Then 25 μL Et_3N was injected to the flask and stirred for 2 days, the resulting mixture was cooled down to room temperature and concentrated. The residue was purified by GPC with DCM as eluent to afford compound **6** as a red oil (18.0 mg, 29%). ^1H NMR (600 MHz, $CDCl_3$) δ = 9.89 (s, 4H), 9.59 (s, 2H), 8.72 (s, 4H), 1.46 – 1.42 (m, 48H), 1.35 – 1.30 (m, 48H), 1.06 (t, *J* = 7.8 Hz, 24H), 0.93 – 0.82 (m, 36H) ppm. ^{13}C NMR (150 MHz, $CDCl_3$) δ = 180.2, 143.7, 141.8, 141.1, 139.5, 138.9, 131.5, 129.5, 129.5, 33.4, 31.5, 23.8, 22.6, 14.2, 12.2 ppm. MS (APCI-TOF) (*m/z*) $[M+H]^+$ calcd for $C_{110}H_{167}N_4O_4Si_4$ 1720.2059, found

1720.2657.

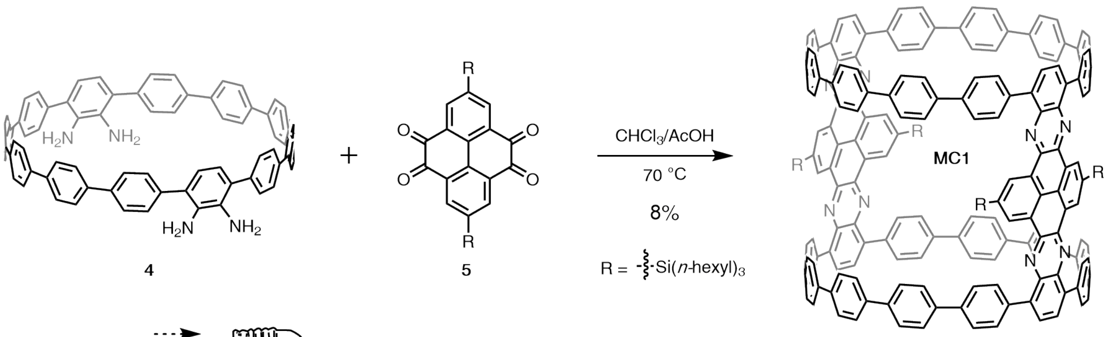

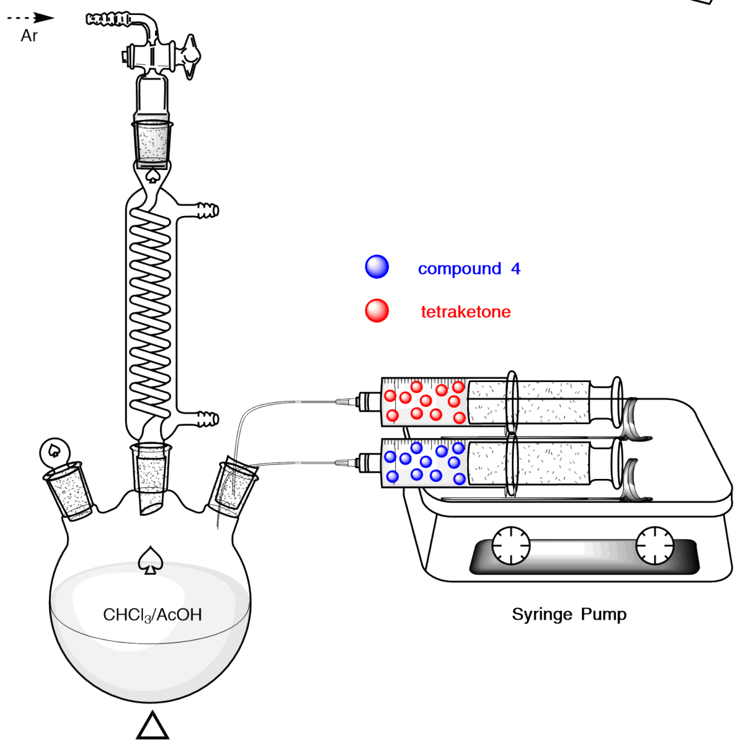

To a 500 mL round bottom flask containing a magnetic stirring bar, 120 mL degassed $CHCl_3$ and 20 mL degassed AcOH were added and heated to 70 °C. Then, compound **4** (20.0 mg, 20.6 μmol) dissolved in 40 mL degassed $CHCl_3$ and compound **5** (16.9 mg, 20.6 μmol) dissolved in 40 mL degassed $CHCl_3$ were added dropwise to the flask through a two-channel syringe pump over 2 h. After stirring for 3 days, the resulting mixture was cooled down to room temperature and concentrated. The residue was purified by column chromatography (alumina, DCM), the flushed fraction was collected and further purified using GPC with DCM as eluent to afford **MC1** as a yellow solid (3.1 mg, 8%). ^{1}H NMR (600 MHz, $CDCl_3$) δ = 9.80 (s, 8H), 8.13 (d, J = 8.6 Hz, 16H), 8.01 (s, 8H), 7.75 – 7.57 (m, 64H), 1.51 – 1.47 (m, 24H), 1.41 – 1.36 (m, 24H), 1.24 – 1.18 (m, 24H), 0.91 – 0.86 (m, 48H), 0.77 – 0.71 (m, 36H) ppm. ^{1}H NMR (600 MHz, CD_2Cl_2) δ = 9.70 (s, 8H), 8.07 (d, J = 8.4 Hz, 16H), 7.97 (s, 8H), 7.66 – 7.51 (m, 64H),

1.51 – 1.42 (m, 24H), 1.20 – 1.14 (m, 48H), 0.88 – 0.79 (m, 48H), 0.75 – 0.69 (m, 36H) ppm. DEPT 135 ^{13}C NMR (150 MHz, $CDCl_3$) δ = 133.0, 131.3, 129.5, 127.4, 127.3, 127.2, 127.0, 127.0, 126.4, 33.3, 31.3, 23.8, 22.4, 13.9, 12.4 ppm. HRMS (MALDI-TOF) *m/z* $[M+H]^+$ calcd for $C_{248}H_{253}N_8Si_4$ 3454.9115, found 3454.9168.

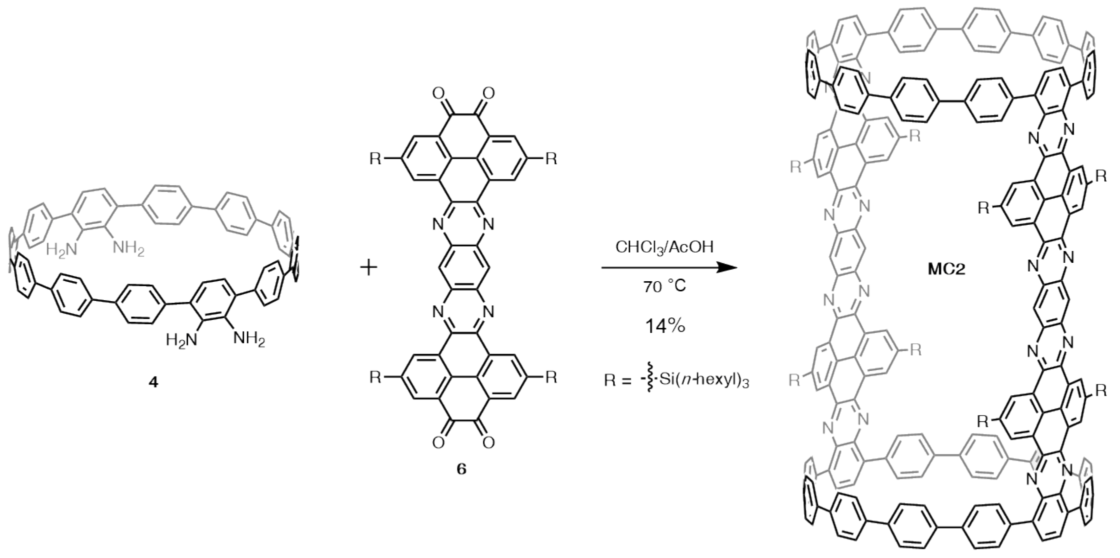

To a 500 mL round bottom flask containing a magnetic stirring bar, 120 mL degassed $CHCl_3$ and 20 mL degassed AcOH were added and heated to 70 °C. Then, compound **4** (20.0 mg, 20.6 μmol) dissolved in 40 mL degassed $CHCl_3$ and compound **6** (35.3 mg, 20.6 μmol) dissolved in 40 mL degassed $CHCl_3$ were added dropwise to the flask through a two-channel syringe pump over 2 h. After stirring for 3 days, the resulting mixture was cooled down to room temperature and concentrated. The residue was purified by column chromatography (alumina, DCM), the flushed fraction was collected and further purified using GPC with DCM as eluent to afford **MC2** as a red solid (7.2 mg, 14%). ^{1}H NMR (600 MHz, THF-d_8) δ = 10.07 (s, 8H), 9.95 (s, 8H), 9.54 (s, 4H), 8.20 (d, J = 7.7 Hz, 16H), 8.01 (s, 8H), 7.73 – 7.62 (m, 64H), 1.64 – 1.56 (m, 48H), 1.51 – 1.44 (m, 48H), 1.35 – 1.29 (m, 48H), 1.26 – 1.20 (m, 96H), 0.89 – 0.81 (m, 72H) ppm. ^{13}C NMR (150 MHz, $CDCl_3$) δ = 141.3, 141.2, 141.0, 140.9, 139.7, 139.4, 139.3, 138.6, 138.5, 137.6, 137.2, 134.5, 133.8, 132.4, 131.5, 130.7, 129.7, 129.1, 128.8, 128.7, 127.7, 127.3, 127.2, 127.2, 33.6, 31.5, 24.0, 22.7, 14.2, 12.6 ppm. DEPT 135 ^{13}C NMR (150 MHz, $CDCl_3$) δ = 134.3, 133.6, 131.3, 130.5, 129.5, 128.6, 127.5, 127.1, 127.0, 127.0, 33.4, 31.3, 23.8, 22.5, 14.0, 12.4 ppm. HRMS (MALDI-TOF) *m/z* $[M+H]^+$ calcd for $C_{364}H_{421}N_{16}Si_8$ 5240.1584, found 5240.1512.

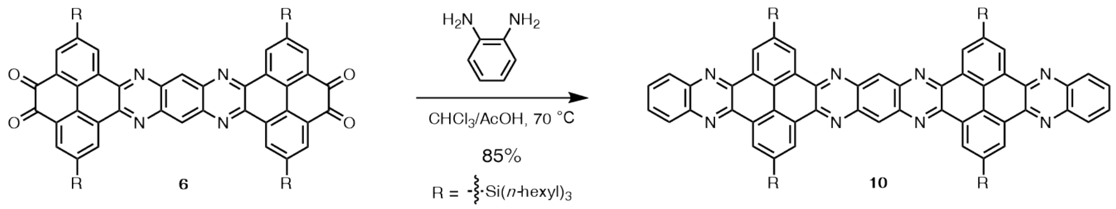

o-Phenylenediamine (1.9 mg, 17.4 μmol) and compound **6** (12.0 mg, 7.0 μmol) were added to a 10 mL Schlenk flask containing a magnetic stirring bar, and 2 mL degassed $CHCl_3$ and 0.5 mL acetic acid were injected to the flask and heated to 70 °C. After stirred for 2 h, the resulting mixture was cooled down to room temperature and poured into 10 mL methanol. The precipitate was then filtered and washed with 2 mL methanol to afford compound **10** as a red solid (11.0 mg, 85%). ^{1}H NMR (600 MHz, $CDCl_3$) δ = 10.00 (s, 4H), 9.95 (s, 4H), 9.55 (s, 2H), 8.44 (s, 4H), 7.91 (s, 4H), 1.63 – 1.57 (m, 24H), 1.55 – 1.50 (m, 24H), 1.43 – 1.33 (m, 48H), 1.23 (t, 24H), 0.92 (t, *J* = 6.8 Hz, 36H) ppm. ^{13}C NMR (150 MHz, $CDCl_3$) δ = 180.2, 143.7, 141.8, 141.1, 139.5, 138.9, 131.5, 129.5, 129.5, 33.4, 31.5, 23.8, 22.6, 14.2, 12.2 ppm. MS (MALDI-TOF) (*m/z*) $[M+H]^+$ calcd for $C_{122}H_{175}N_8Si_4$ 1864.3016, found 1864.6151.

2. Structural analysis

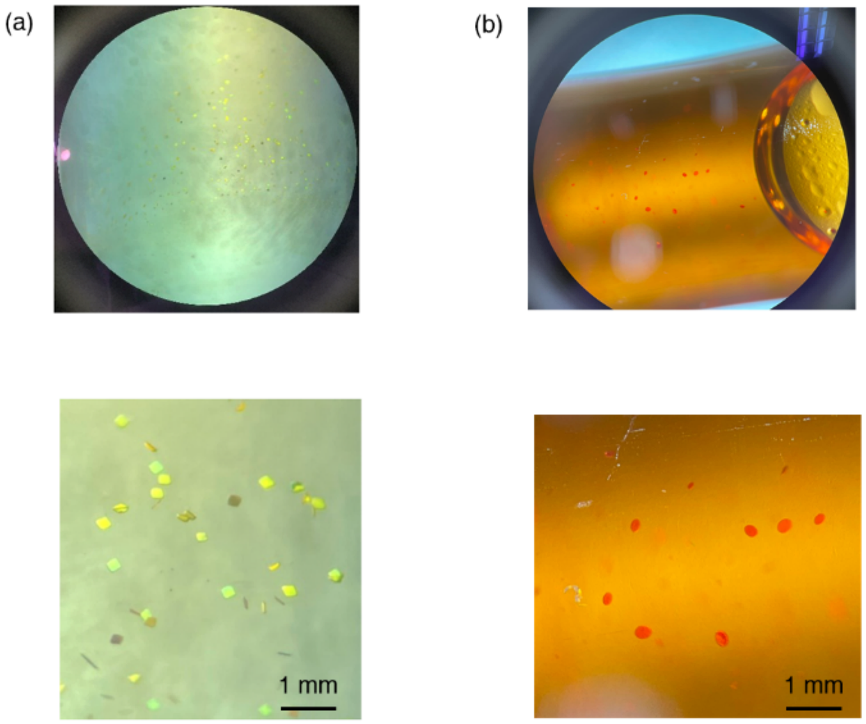

Figure S1. Pictures of Single crystals of **MC1** (a) and **MC2** (b) taken by OPTEX SZ680.

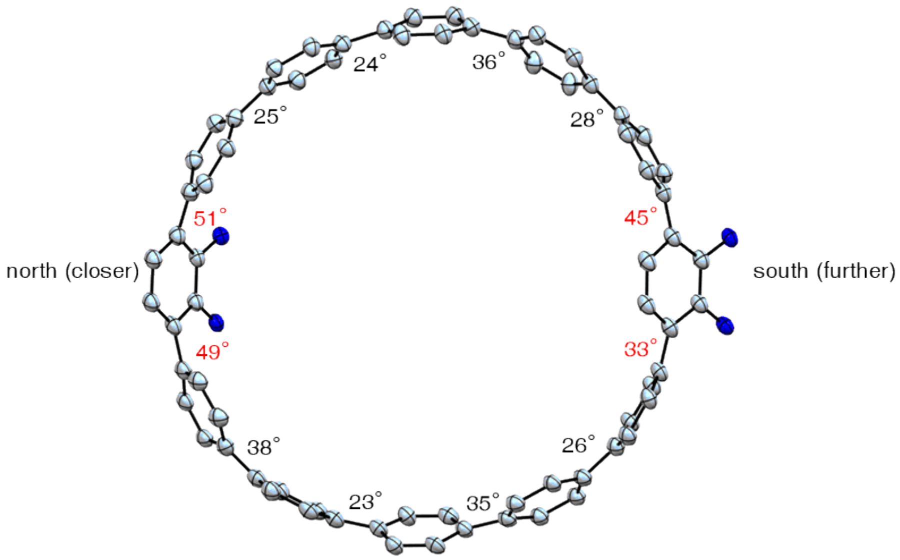

Figure S2. The torsional angles of the aryl rings on the CPP part for **MC1** (ORTEP drawing in top view with thermal ellipsoids set to 12% probability).

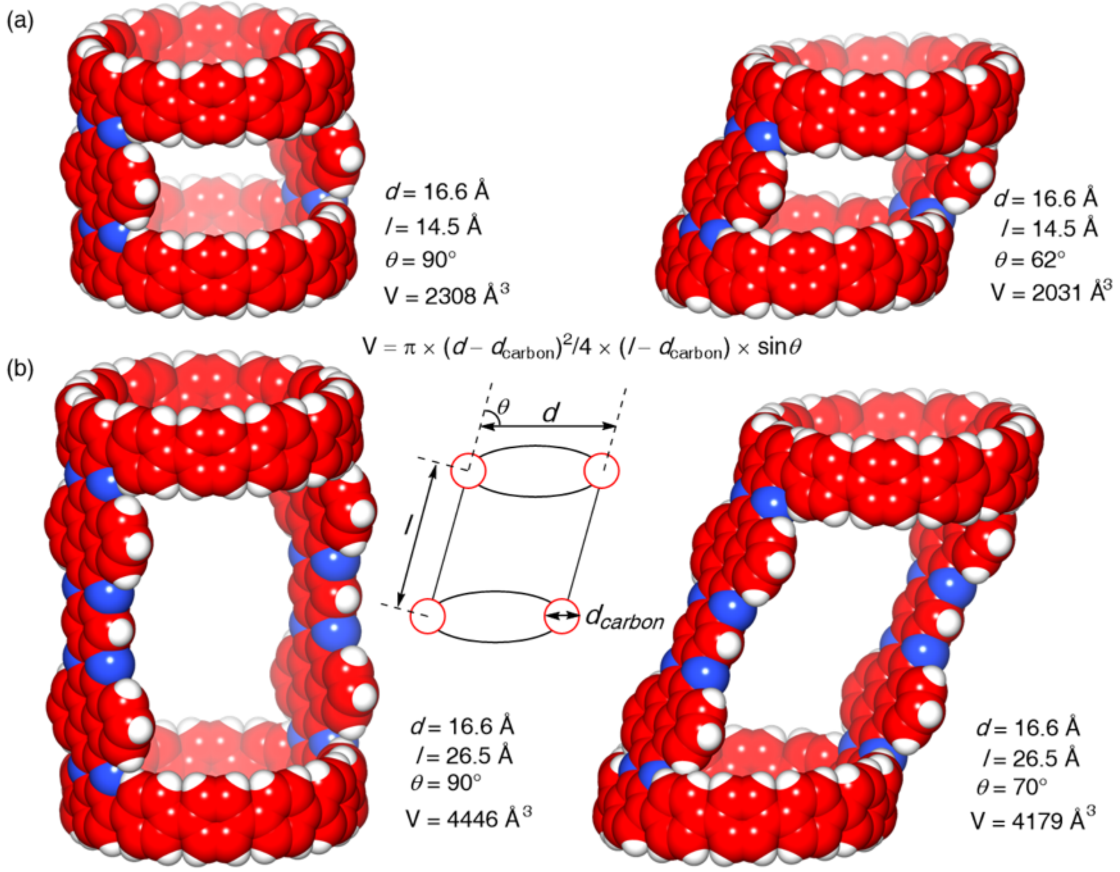

Figure S3. Calculated volume for **MC1** (a) and **MC2** (b) from ideal structures.

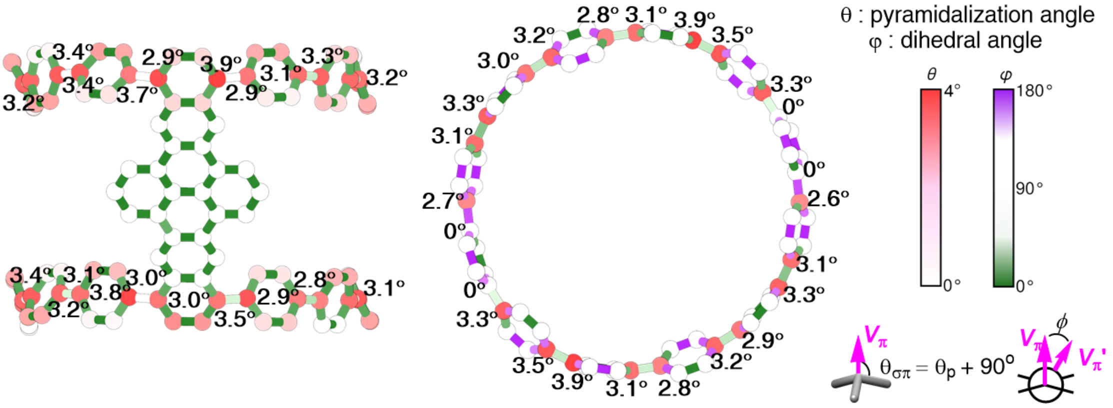

Figure S4. POAV analysis for crystal structures of **MC1** and [12]CPP.

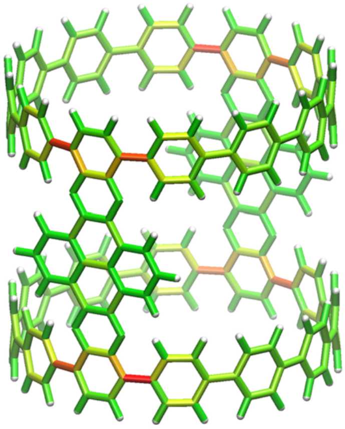

Total : 281 kcal/mol

Figure S5. Strainvis analysis for **MC1** with D_{2h}-symemtric structure.

Table S1. Crystal data and structure refinement for **MC1** at 95 K.

Data deposition	CCDC 2363037
Empirical formula	$C_{285.86}H_{340.34}N_8Si_4$
Formula weight	4000.66
Temperature/K	95(2)
Crystal system	monoclinic
Space group	$P2_1/c$
Unit cell dimensions	*a* = 19.117(16) Å *α* = 90°
	b = 19.984(10) Å *β* = 102.54(3)°
	c = 32.685(17) Å *γ* = 90°
Volume	12189(13) Å^3
Z	2
ρ_{calc}g/cm^3	1.090
μ/mm^{-1}	0.144
F(000)	4335
Crystal size/mm^3	0.02× 0.02×0.01
Radiation	synchrotron (wavelength = 0.9000)
Theta range for data collection/°	1.382 to 27.800
Index ranges	-19 ≤ *h* ≤ 19, -20 ≤ k ≤ 20, -33 ≤ *l* ≤ 33
Reflections collected	181918
Independent reflections	13808 [R_{int} = 0.0695, R_{sigma} = 0.0261]
Data/restraints/parameters	13808/1337/1822
Goodness-of-fit on F^2	1.101
Final *R* indices [I>2σ(I)]	R_1 = 0.0960, wR_2 = 0.2681
R indices (all data)	R_1 = 0.1490, wR_2 = 0.3259
Largest diff. peak/hole / e Å^{-3}	0.352/-0.223

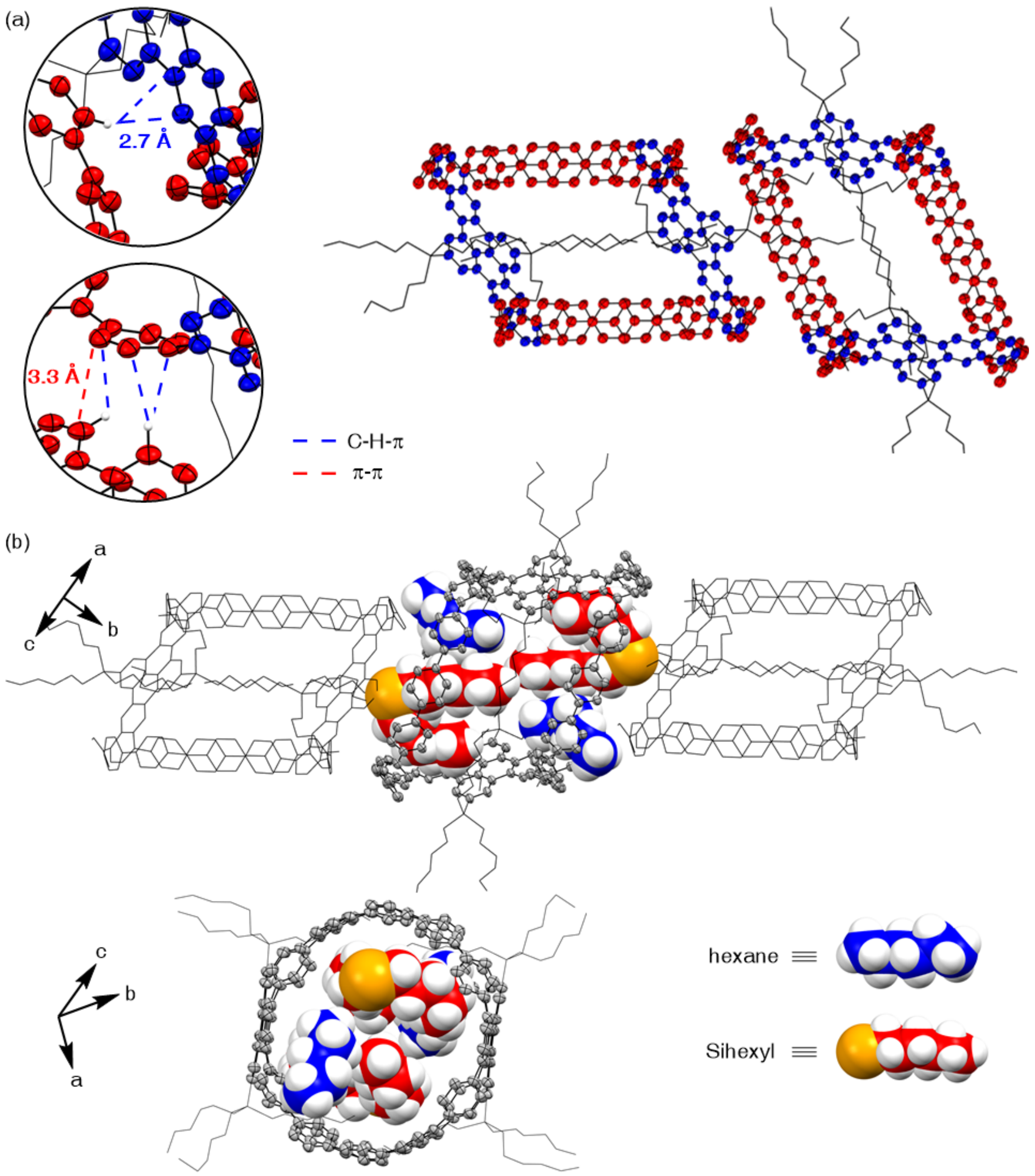

Figure S6. (a) Packing mode of **MC1**. (b) Illustration of two alkyl chain and solvent molecules filled up the cavity of the central molecule. Representative hydrogen atoms and solvent molecules are shown while others are omitted for clarity.

(a)

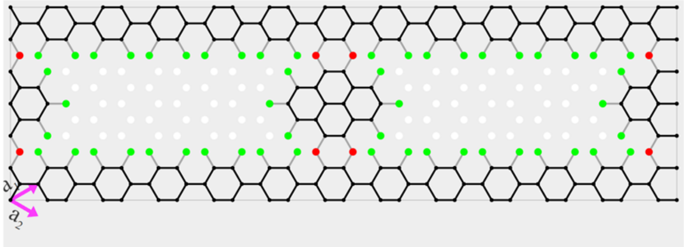

chiral vector (n, m) = (12, 12)
length index (t_{l}) = 6
bond filling index (F_{b}) = 206/444
46.40% (C-C bonds)
atom filling index (F_{a}) = 176/312
56.41% (sp^2-carbon)

coordinates of ● (Atomic number = 7) :

(5/3, −4/3), (7, 4), (10, 1), (13, 10), (14/3, −13/3), (16, 7), (23/3, 14/3), (32/3, 5/3)

(b)

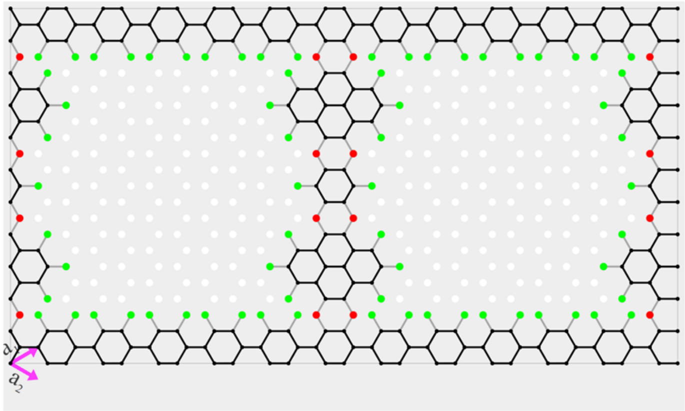

chiral vector (n, m) = (12, 12)
length index (t_{l}) = 11
bond filling index (F_{b}) = 256/804
31.84% (C-C bonds)
atom filling index (F_{a}) = 220/552
39.86% (sp^2-carbon)

coordinates of ● (Atomic number = 7) :

(5/3, −4/3), (7, 4), (10, 1), (12, −1), (13, 10), (14/3, −13/3), (15, −4), (16, 7), (18, 5), (20/3, −19/3), (21, 2), (23/3, 14/3), (29/3, −28/3), (32/3, 5/3), (38/3, −1/3), (47/3, −10/3)

Figure S7. Geometric measures of **MC1** and **MC2**.

3. Solution phase structural characterization

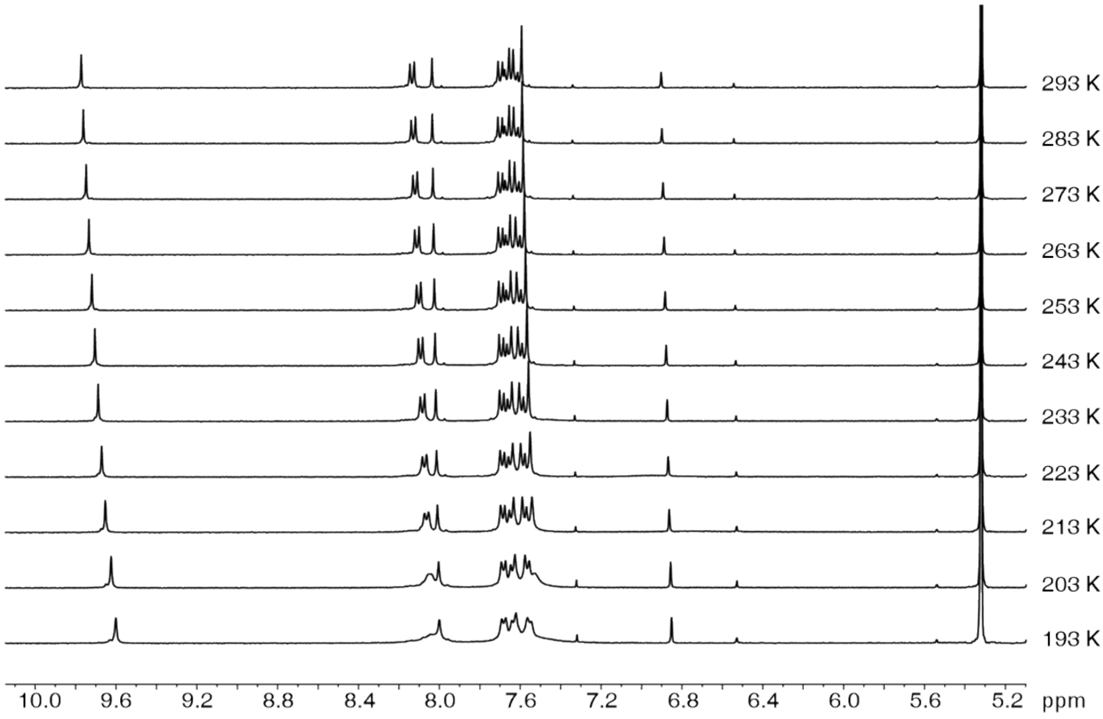

Figure S8. Variable-temperature ^{1}H NMR spectra of **MC1** at the aromatic region (dichloromethane-d_2/CS_2, 600 MHz).

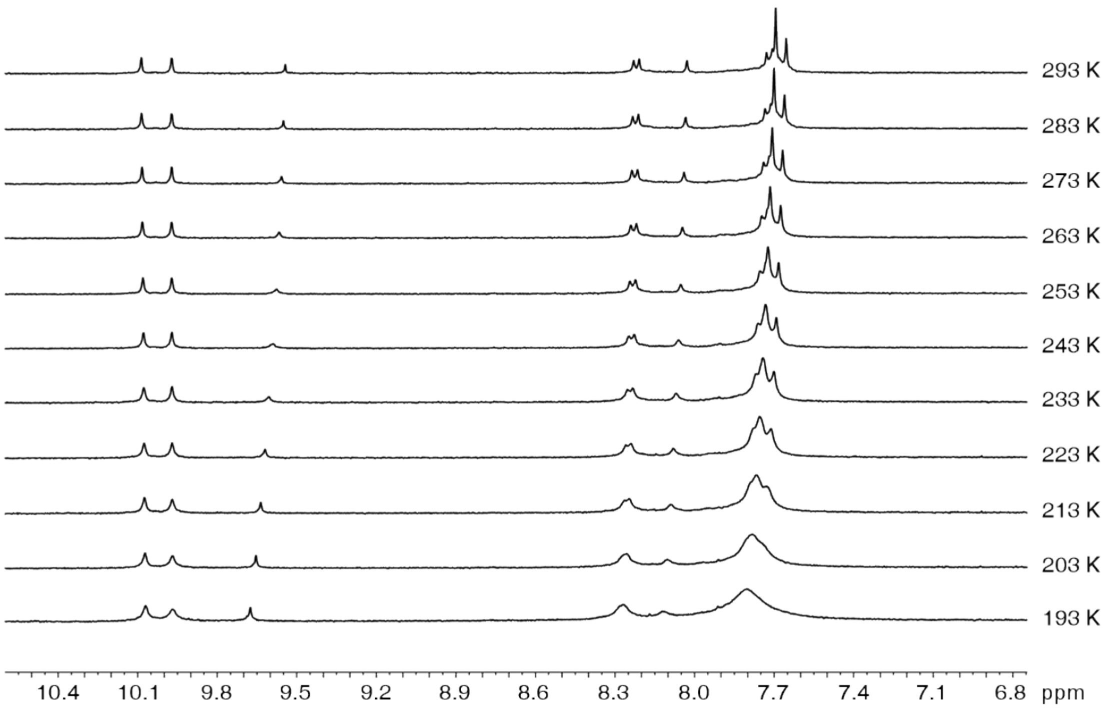

Figure S9. Variable-temperature ^{1}H NMR spectra of **MC2** at the aromatic region (terotetrahydrofuran-d_8/CS_2, 600 MHz).

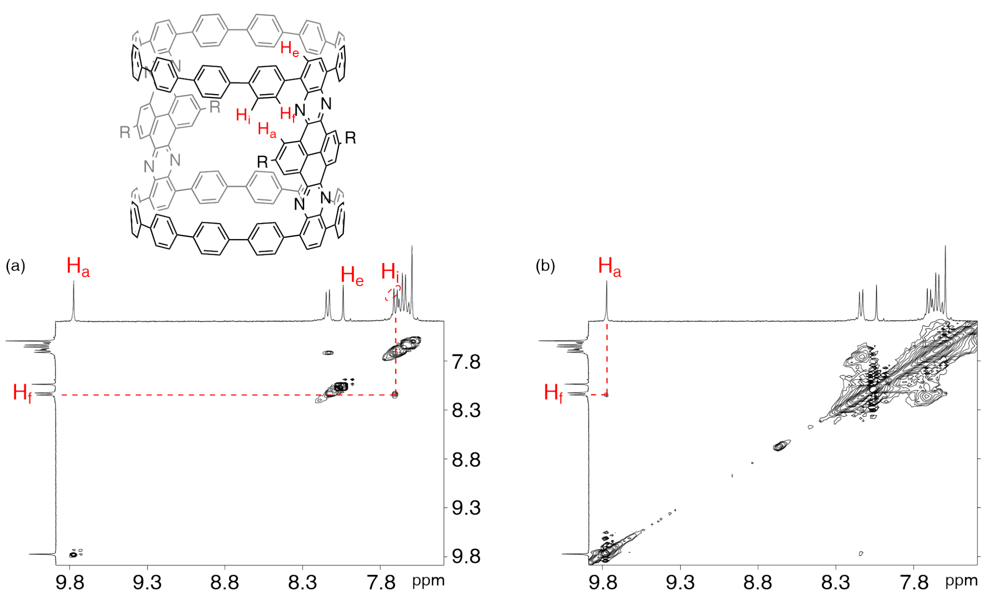

Figure S10. Assignments of ^{1}H NMR resonances of **MC1**. Spectra were taken in dichloromethane-d_2/CS_2 at 298 K. (a) COSY spectrum. (b) NOESY spectrum.

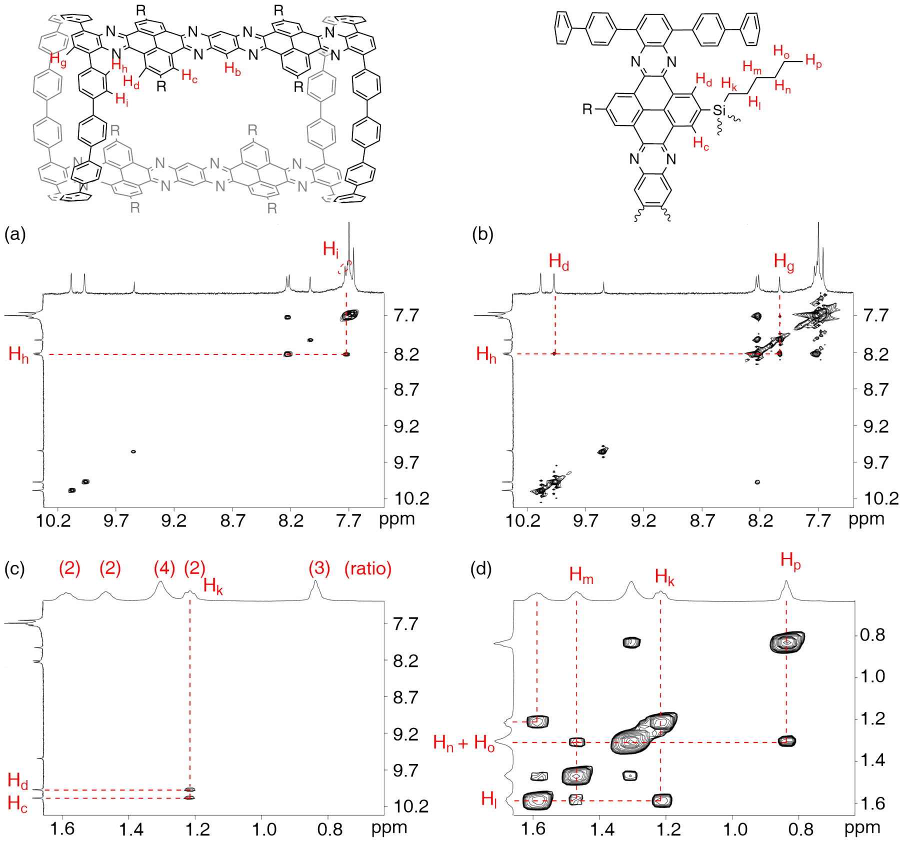

Figure S11. Assignments of ^{1}H NMR resonances of **MC2**. Spectra were taken in tetrahydrofuran-d_8/CS_2 at 298 K. (a) (d) COSY spectrum. (b) (c) NOESY spectrum.

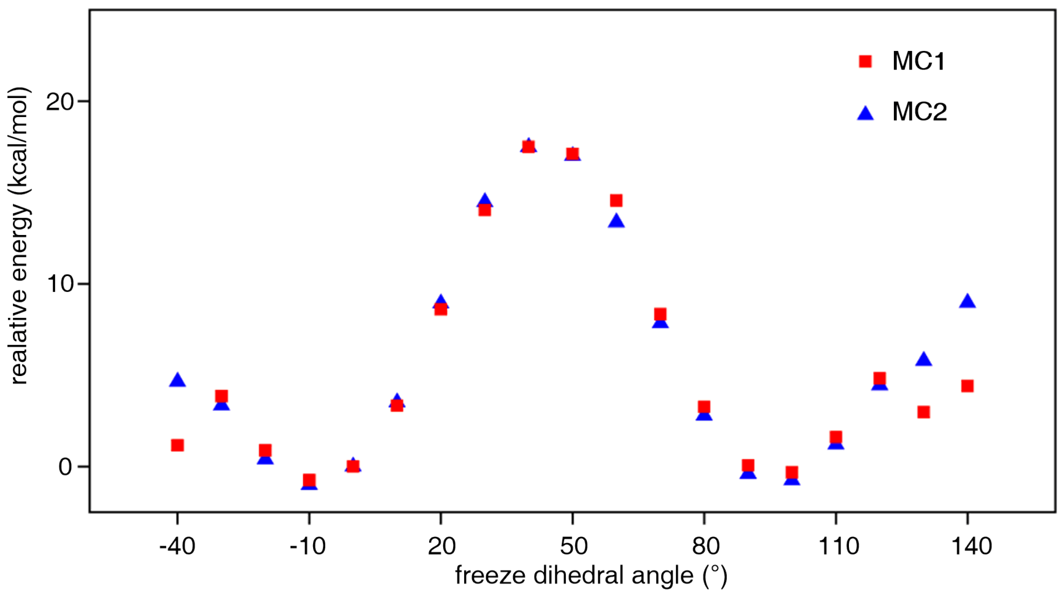

Figure S12. Energy profile of the swinging motion of **MC1** and **MC2** determined by relaxed scan analysis at PM6 level.

4. Photophysical properties

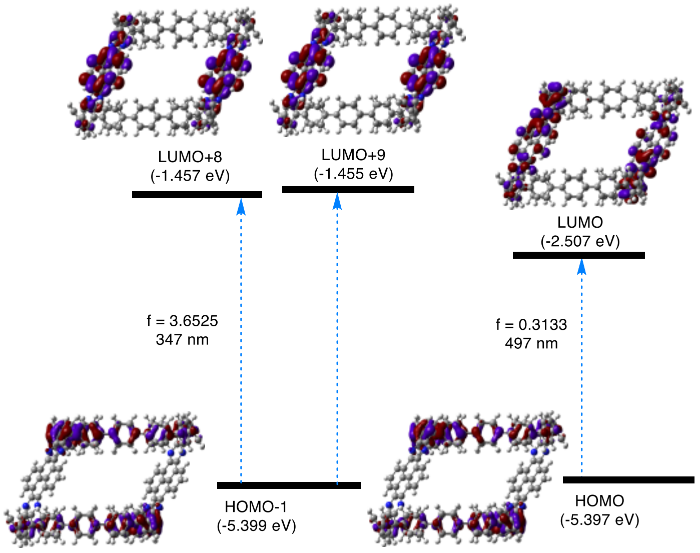

Figure S13. Molecular orbitals and energy diagram of **MC1** from TD-DFT calculation.

Table S2. Calculated electronic transitions for **MC1** without substituents.

No.	Wavelength (nm)	Oscillator Strength	Major Contributions
1	498.10	0.0000	H-1→L (40%), H→L+1 (31%), H→L+2 (9%)
2	497.35	0.3133	H→L (44%), H→L+1 (28%), H-1→L+2 (10%)
3	480.87	0.1558	H-1→L+1 (32%), H→L+3 (15%), H→L (15%), H-1→L+2 (10%)
4	437.10	0.2029	H-3→L+1 (52%), H-2→L (25%), H-2→L+3 (7%)
5	368.96	0.1992	H→L+6 (37%), H-1→L+7 (31%), H-8→L+3 (5%)
6	366.38	0.5815	H-10→L (21%), H-7→L (13%), H-1→L+4 (24%), H→ L+5 (24%)
7	347.90	3.6525	H-1→L+9 (32%), H-1→L+8 (32%), H-2→L+6 (14%)
8	329.33	0.5437	H-2→L+6 (26%), H-1→L+8 (16%), H-3→L+7 (15%), H-5→L+6 (12%)

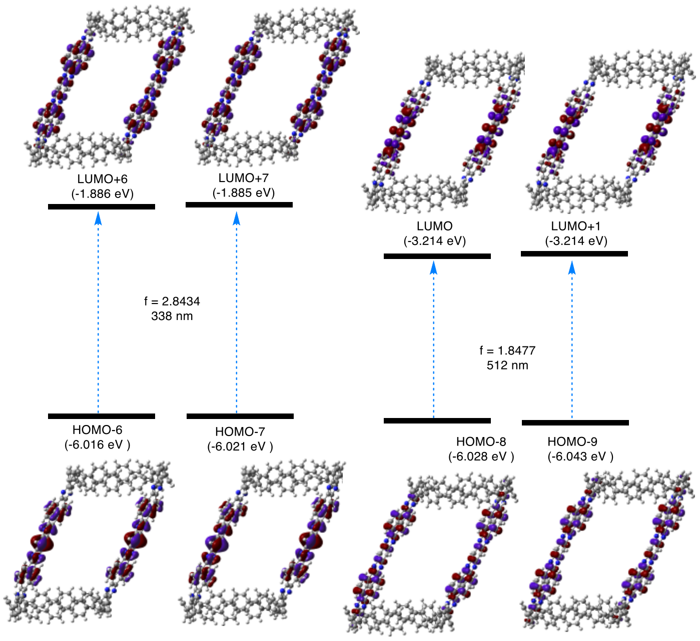

Figure S14. Molecular orbitals and energy diagram of **MC2** from TD-DFT calculation.

Table S3. Calculated electronic transitions for **MC2** without substituents.

No.	Wavelength (nm)	Oscillator Strength	Major Contributions
1	632.66	0.0001	H→L (49%), H-1→L (26%), H→L+1 (23%)
2	513.39	1.1818	H-1→L+3 (29%), H→L+2 (29%), H-9→L+1 (15%), H-8 →L (15%)
3	512.25	1.8477	H-8→L (32%), H-9→L+1 (32%), H→L+2 (14%), H-1→ L+3 (14%)
4	494.03	0.4109	H-1→L+4 (35%), H→L+5 (35%), H-5→L+4 (7%), H-4→ L+5 (7%)
5	451.58	0.2590	H-3→L+4 (25%), H-2→L+5 (22%), H-2→L+2 (19%), H- 3→L+3 (14%)
6	444.49	0.1275	H-5→L+4 (33%), H-4→L+5 (32%), H→L+5 (11%), H-1 →L+4 (11%)
7	401.86	0.4665	H-8→L+3 (35%), H-9→L+2 (27%), H→L+7 (3%), H-1→ L+6 (3%)
8	383.50	0.6517	H→L+7 (22%), H-1→L+6 (20%), H-7→L+6 (26%)
9	338.14	2.8434	H-6→L+7 (27%), H-7→L+6 (26%), H-5→L+6 (9%)

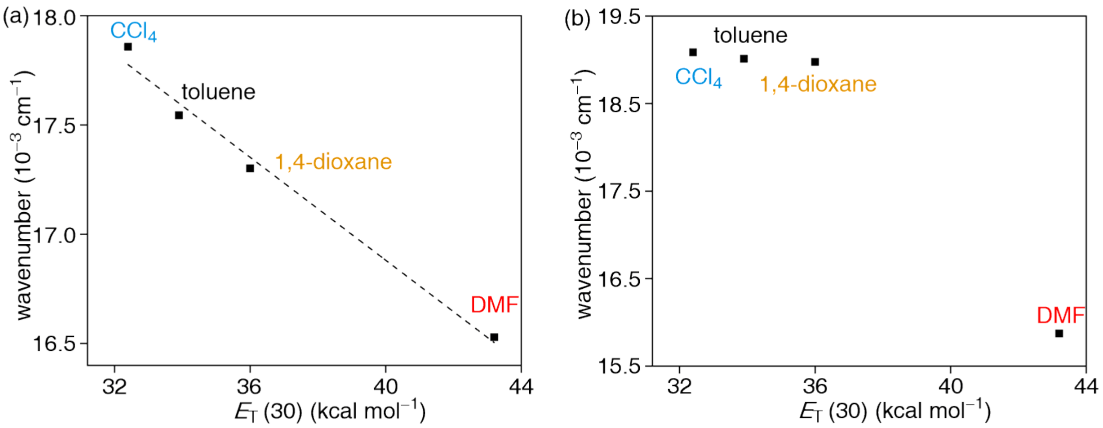

Figure S15. A plot of emission maxima (wavenumber) of **MC1** (a) and **MC2** (b) in various solvents against E_T (30).

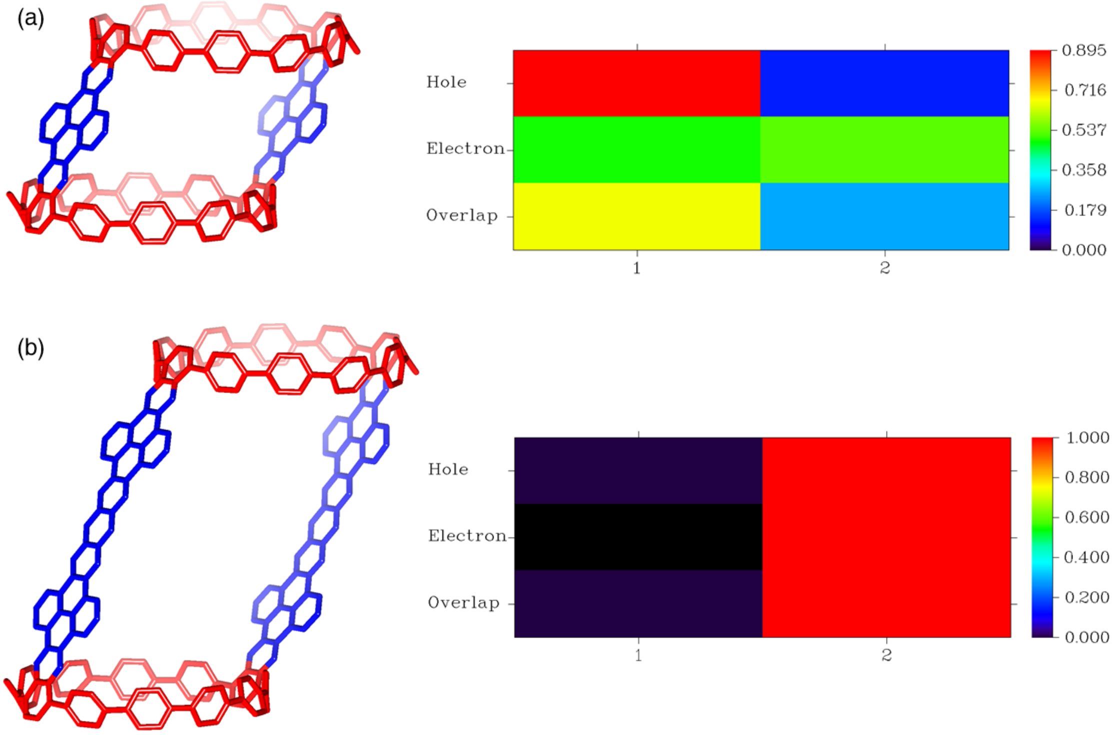

Figure S16. Interfragment charge transfer (IFCT) heat maps based on fragments for the S_1 excited states of **MC1** and **MC2**. Red and blue section indicates fragment 1 (CPP as donor) and fragment 2 (NAM as acceptor), respectively.

Table S4. The calculated charge transfer parameters from hole-electron analysis of **MC1** and **MC2**.

			Hole	Electron	Overlap	CT%	LE%
**MC1**	S_1	Fragment 1	89%	49%	66%	41%	59%
		Fragment 2	13%	53%	26%		
	S_2	Fragment 1	90%	49%	66%	41%	59%
		Fragment 2	13%	53%	26%		
	S_3	Fragment 1	87%	44%	61%	44%	56%
		Fragment 2	16%	59%	30%		
	S_4	Fragment 1	86%	43%	61%	44%	56%
		Fragment 2	17%	60%	31%		
**MC2**	S_1	Fragment 1	0%	0%	0%	0%	100%
		Fragment 2	100%	100%	100%		
	S_2	Fragment 1	0%	0%	0%	0%	100%
		Fragment 2	100%	100%	100%		
	S_3	Fragment 1	3%	1%	1%	2%	98%
		Fragment 2	99%	99%	99%		
	S_4	Fragment 1	3%	0%	1%	3%	97%
		Fragment 2	99%	100%	99%		

(a)

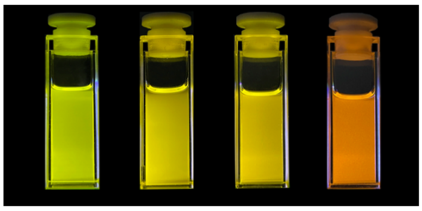

	CCl_4	toluene	dioxane	DMF
Φ =	47%	63%	33%	16%

(b)

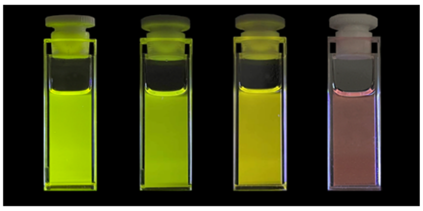

	CCl_4	toluene	dioxane	DMF
Φ =	15%	19%	19%	10%

Figure S17. Photographs of **MC1** (a) and **MC2** (b) under irradiation at 365 nm and the determined absolute quantum yields in different solvents.

5. NMR spectra

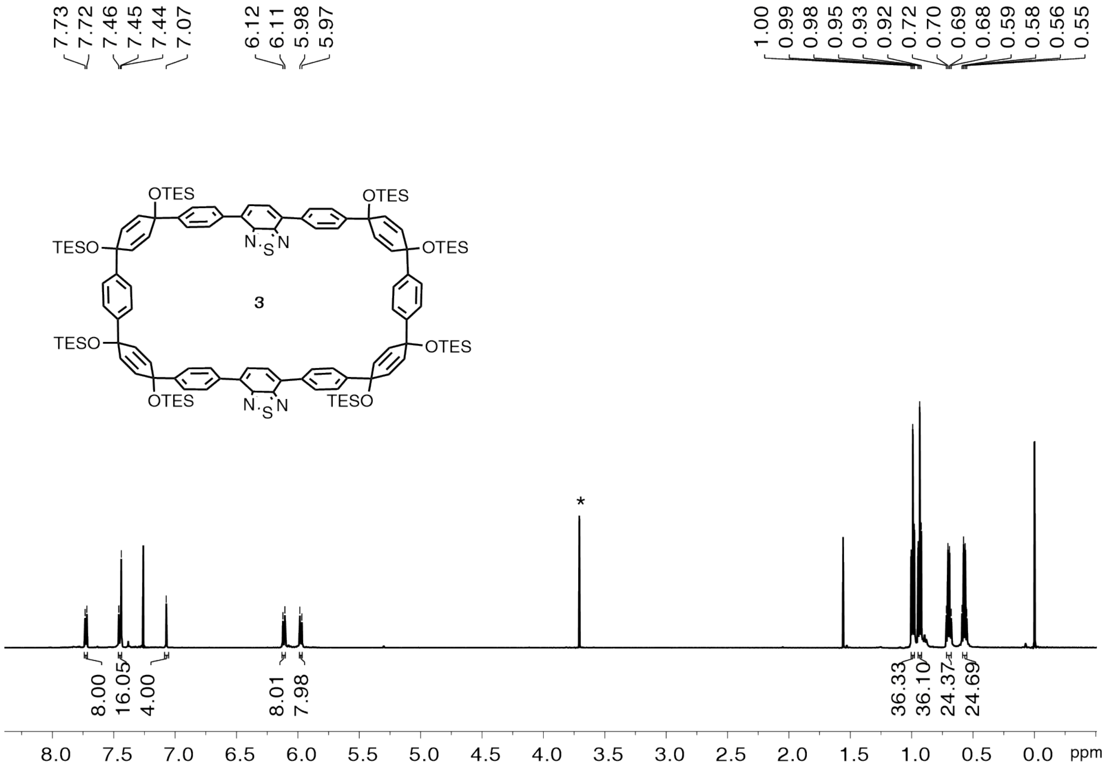

Figure S18. ^{1}H NMR spectrum of **3** in $CDCl_3$ (298 K, 600 MHz). Asterisk indicates signal of solvent.

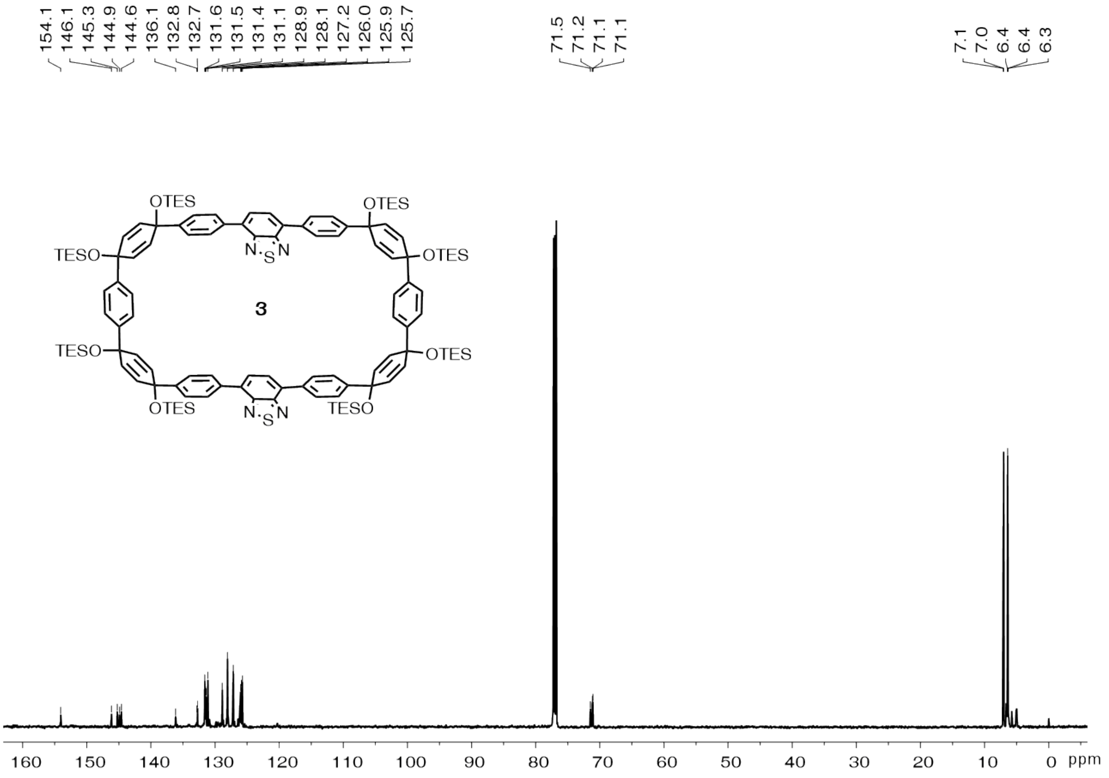

Figure S19. ^{13}C NMR spectrum of **3** in $CDCl_3$ (298 K, 150 MHz).

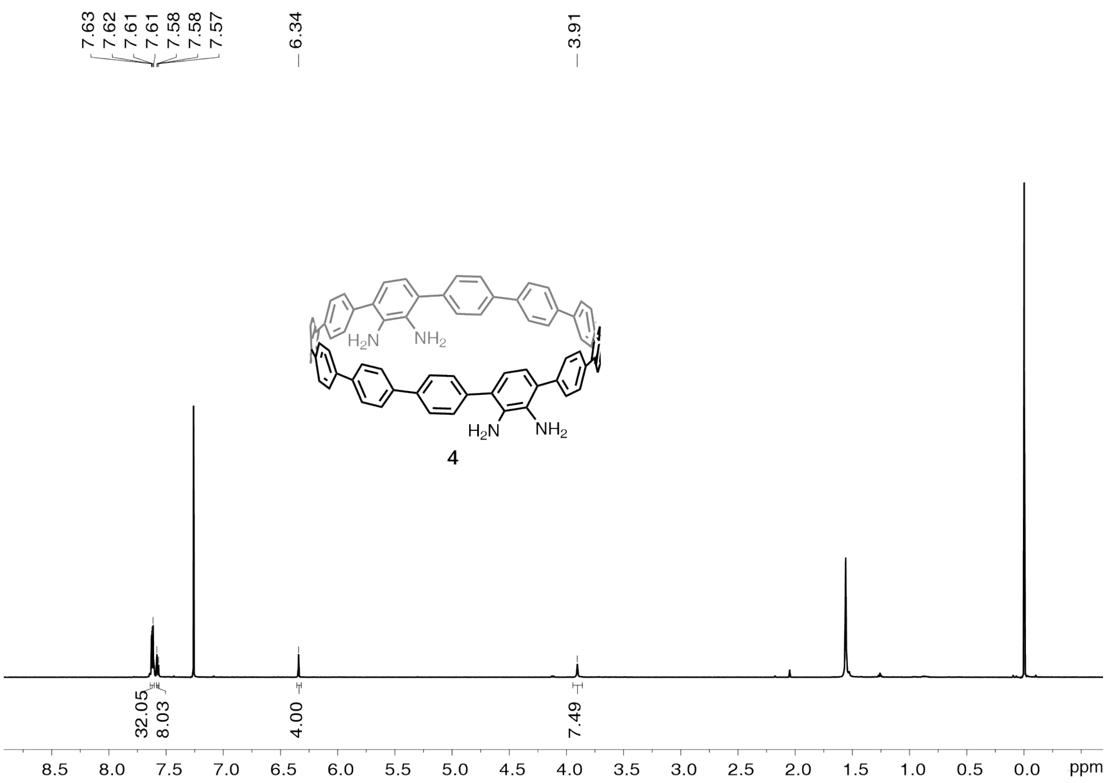

Figure S20. ^{1}H NMR spectrum of **4** in $CDCl_3$ (298 K, 600 MHz).

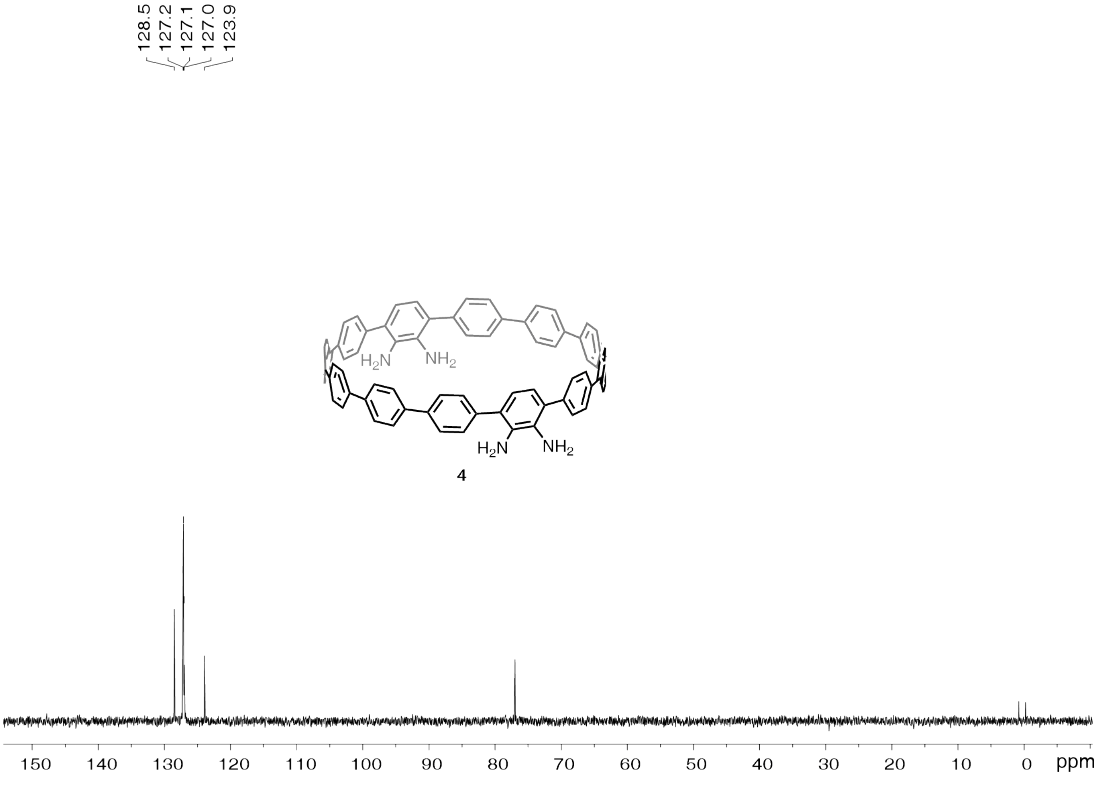

Figure S21. DEPT 135 ^{13}C NMR spectrum of **4** in $CDCl_3$ (298 K, 150 MHz).

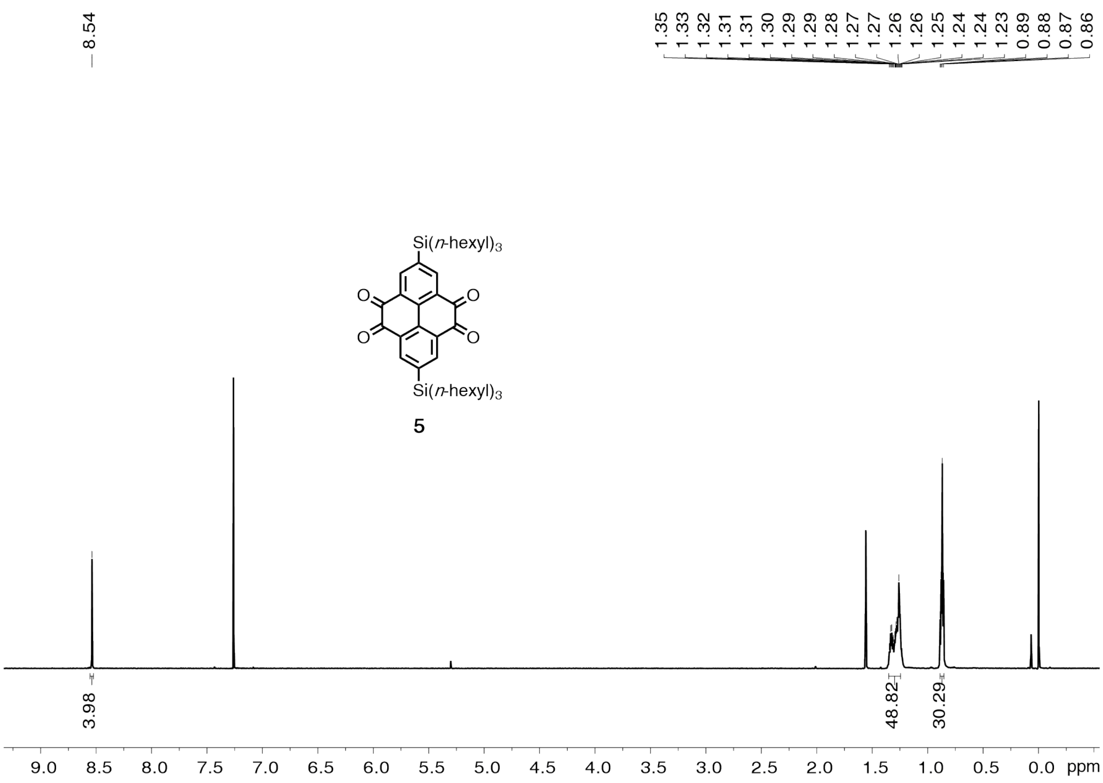

Figure S22. ^{1}H NMR spectrum of **5** in $CDCl_3$ (298 K, 600 MHz).

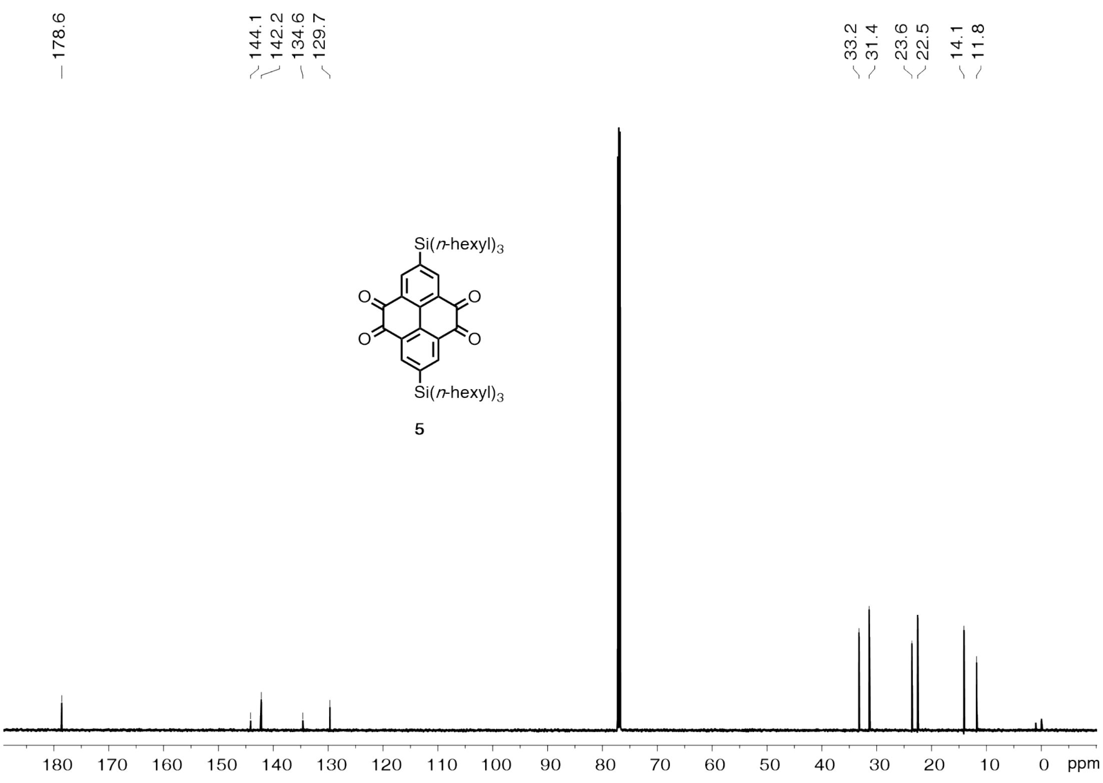

Figure S23. ^{13}C NMR spectrum of **5** in $CDCl_3$ (298 K, 150 MHz).

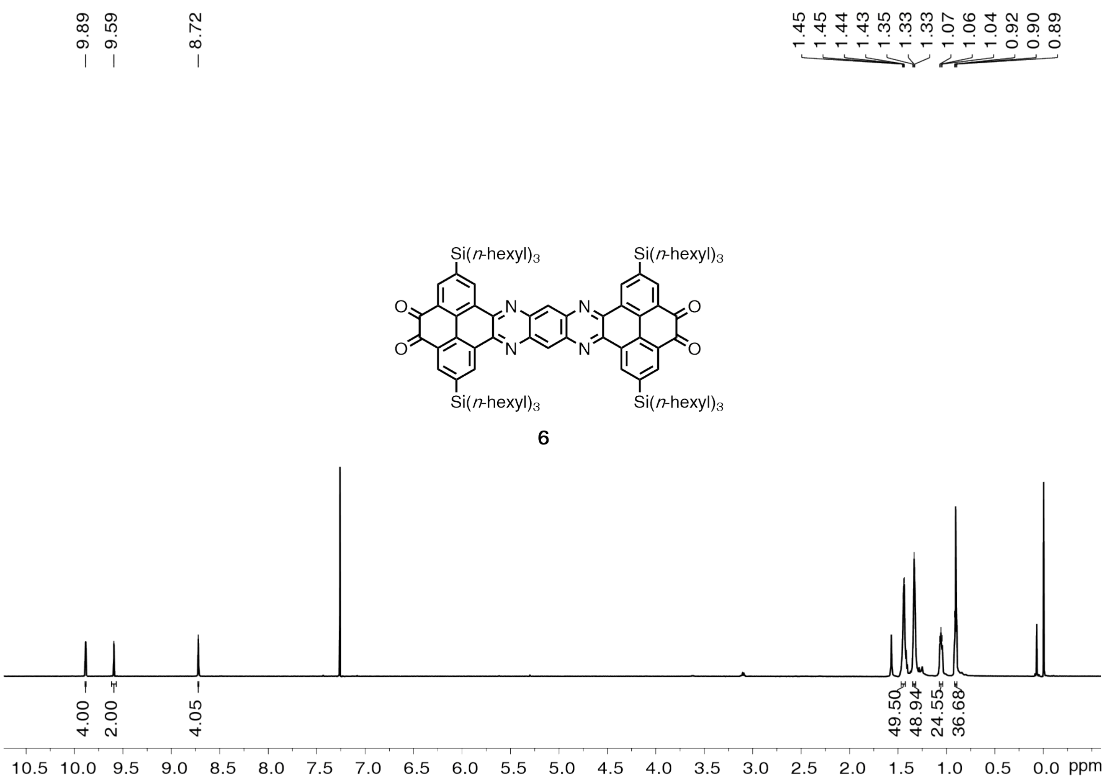

Figure S24. ^{1}H NMR spectrum of **6** in $CDCl_3$ (298 K, 600 MHz).

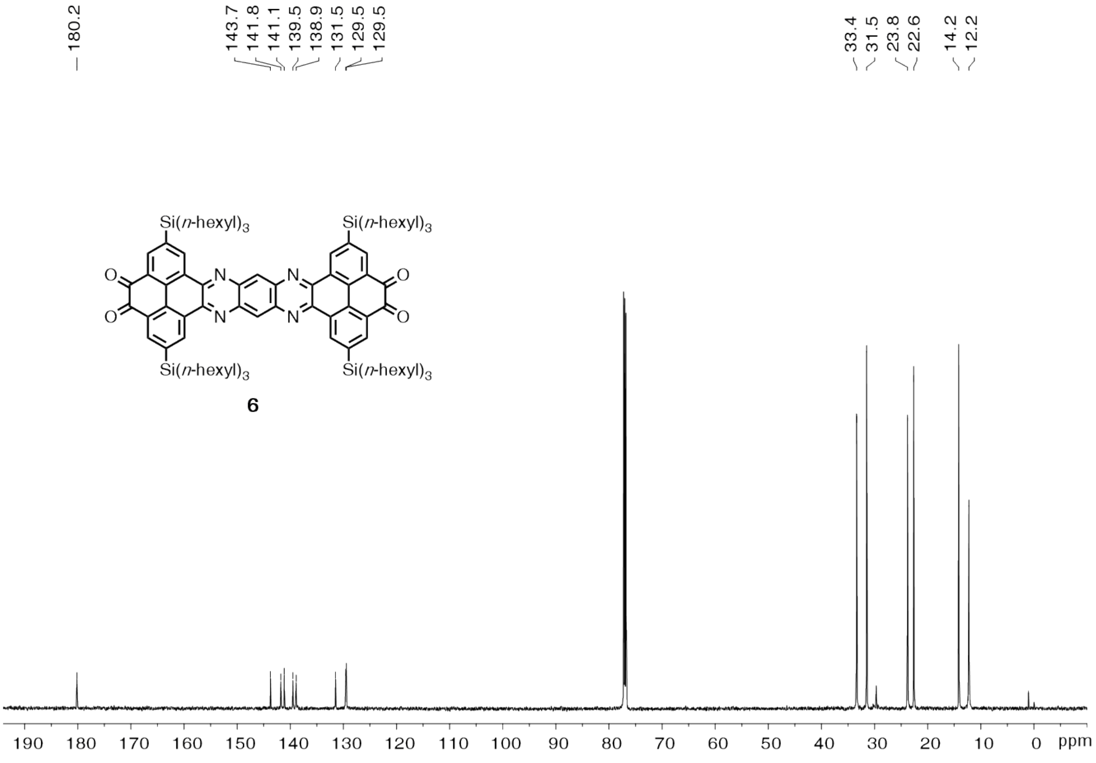

Figure S25. ^{13}C NMR spectrum of **6** in $CDCl_3$ (298 K, 150 MHz).

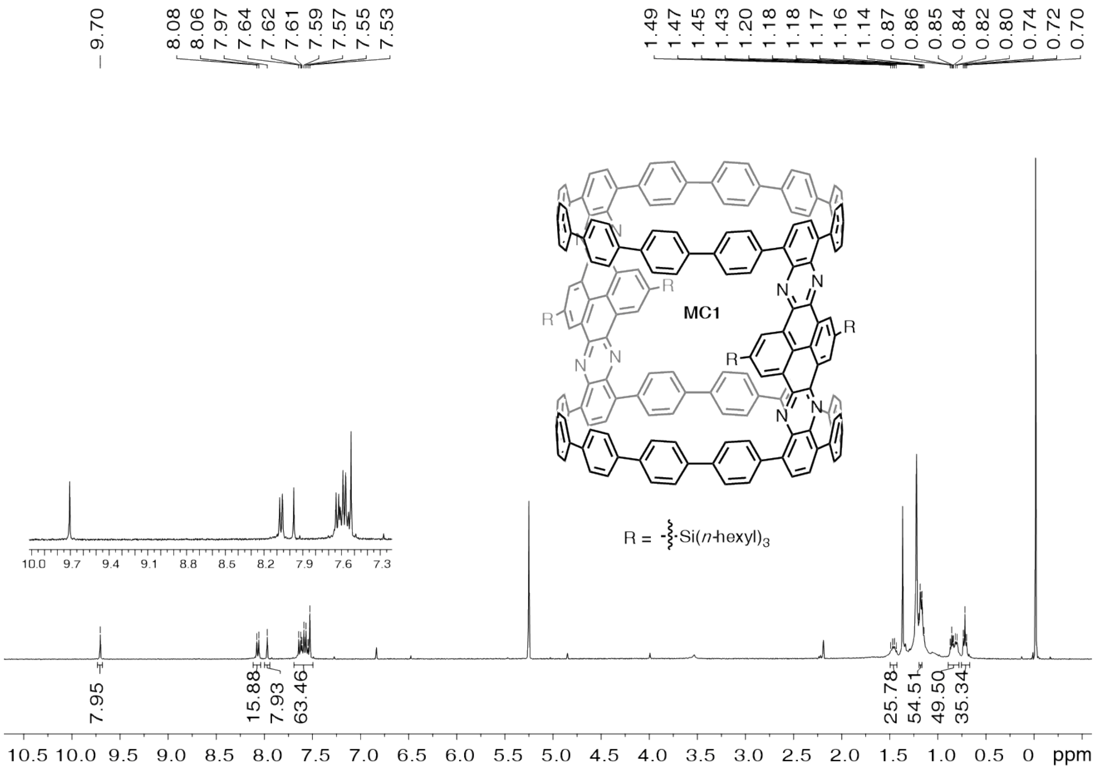

Figure S26. ^{1}H NMR spectrum of **MC1** in CD_2Cl_2, 0.2 mL CS_2 were added (298 K, 150 MHz).

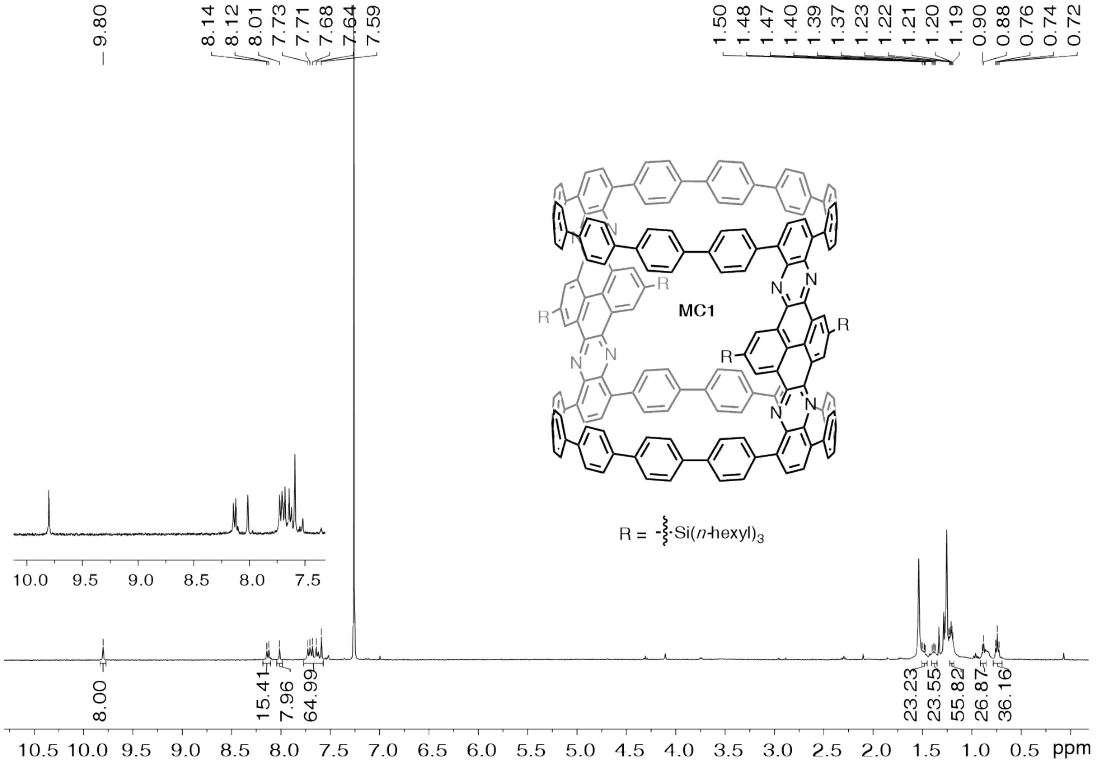

Figure S27. ^{1}H NMR spectrum of **MC1** in $CDCl_3$ (298 K, 150 MHz).

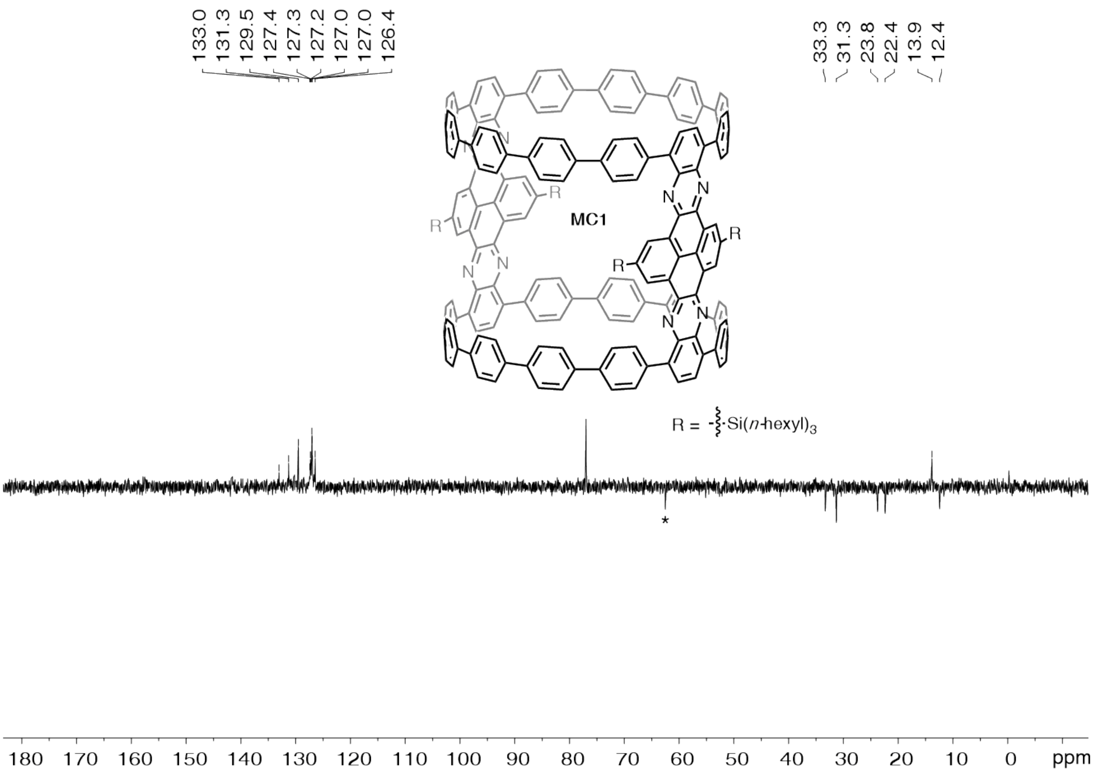

Figure S28. DEPT 135 ^{13}C NMR spectrum of **MC1** in $CDCl_3$ (298 K, 150 MHz). Asterisk indicates signal of solvent.

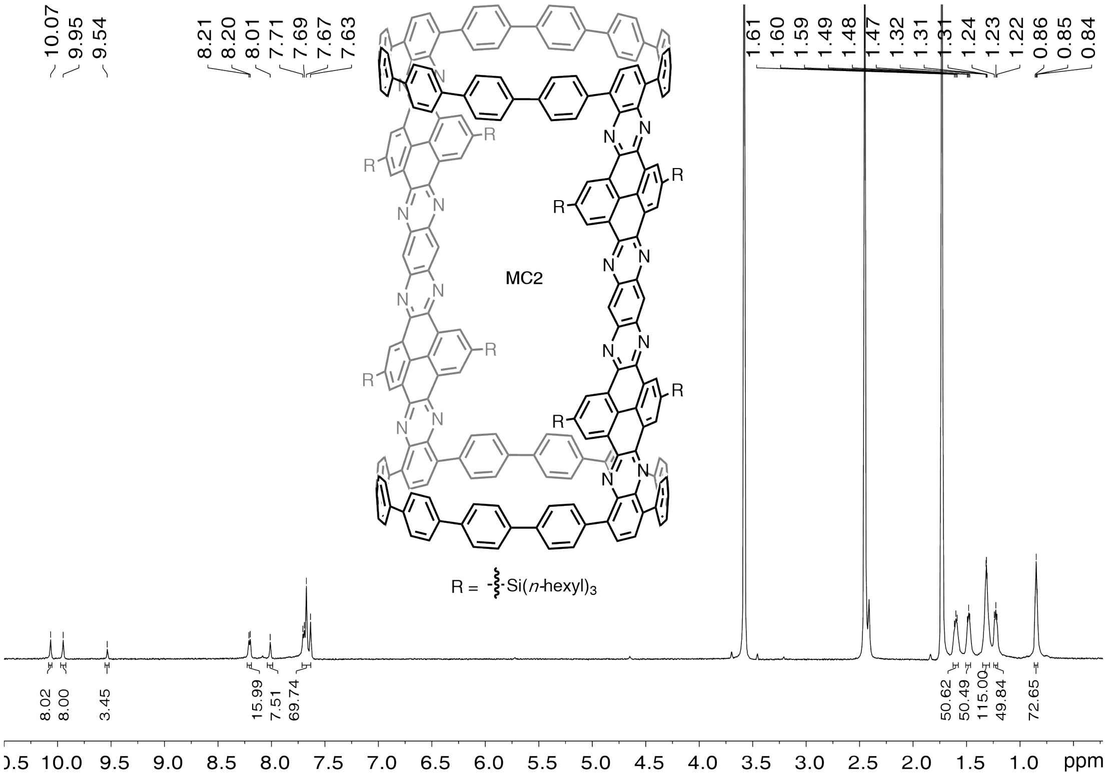

Figure S29. ^{1}H NMR spectrum of **MC2** in THF-d_8, 0.2 mL CS_2 were added (298 K, 150 MHz).

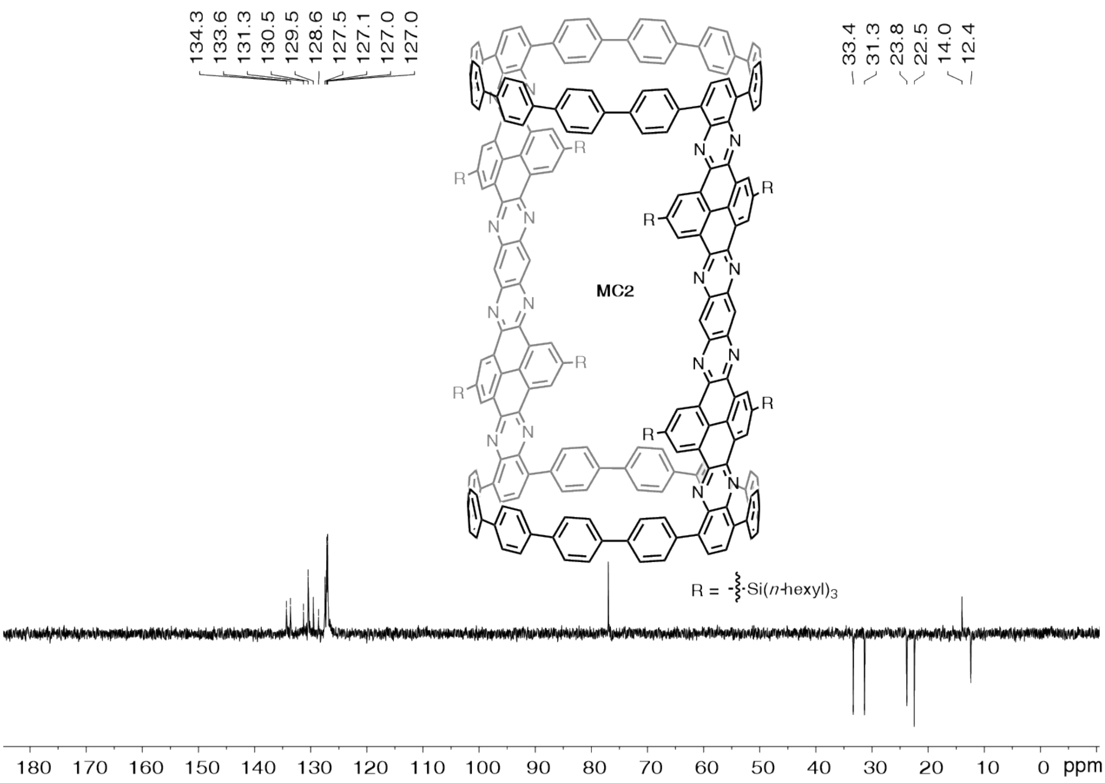

Figure S30. DEPT 135 ^{13}C NMR spectrum of **MC2** in $CDCl_3$ (298 K, 150 MHz).

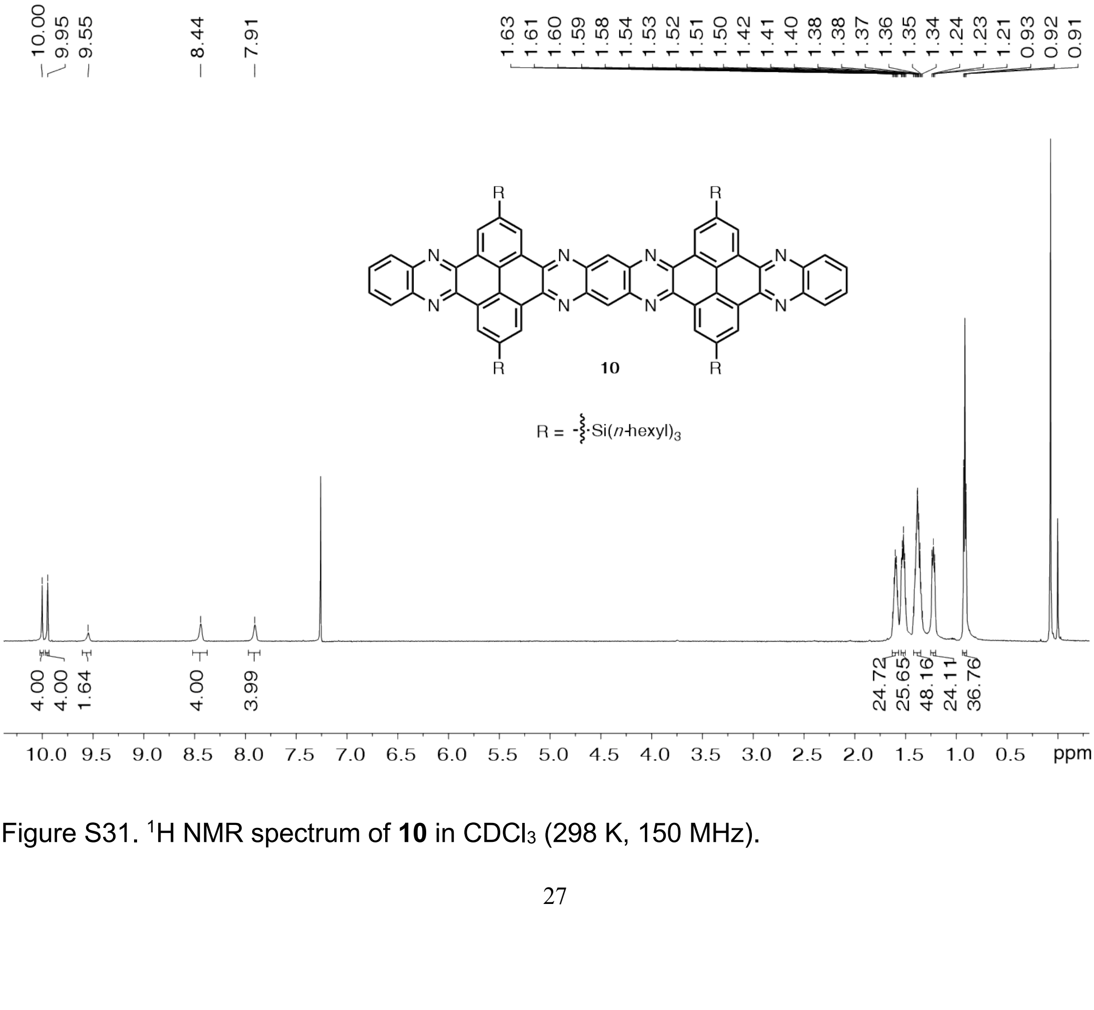

Figure S31. ^{1}H NMR spectrum of **10** in $CDCl_3$ (298 K, 150 MHz).

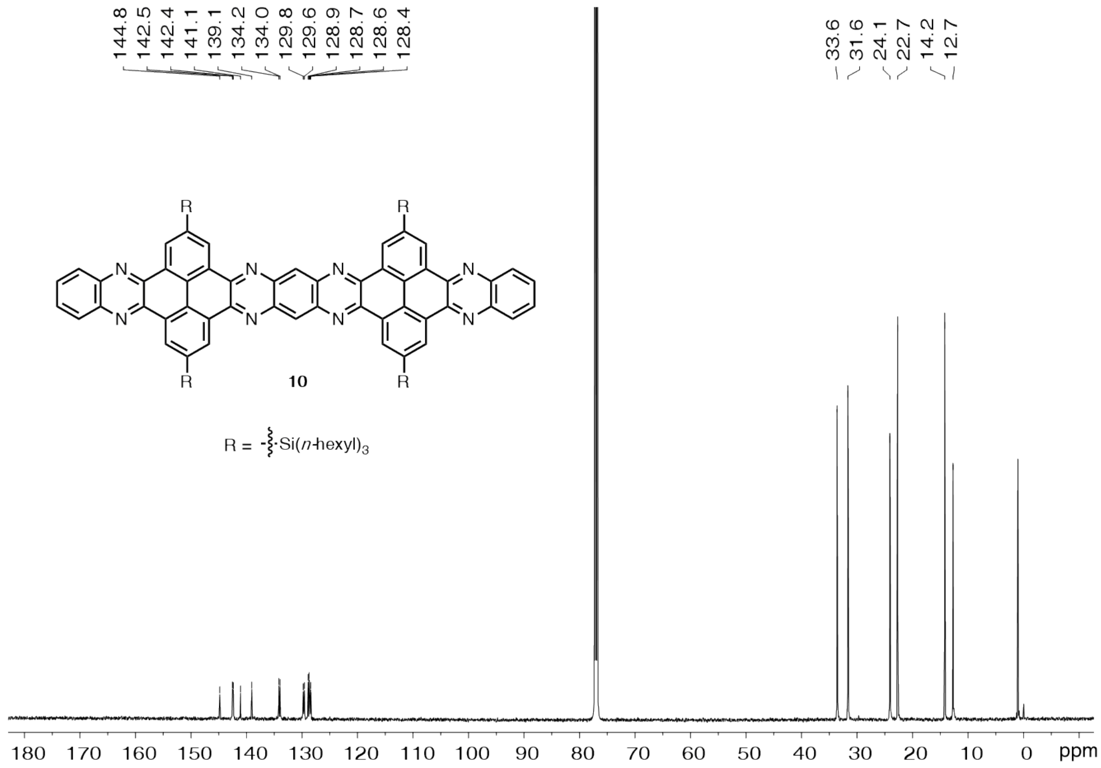

Figure S32. ^{13}C NMR spectrum of **10** in $CDCl_3$ (298 K, 150 MHz).

6. Reference

[1] T. C. Lovell, C. E. Colwell, L. N. Zakharov and R. Jasti, *Chem. Sci.,* 2019, **10**, 3786−3790.

[2] A. G. Crawford, Z. Liu, I. A. I. Mkhalid, M.-H. Thibault, N. Schwarz, G. Alcaraz, A. Steffen, J. C. Collings, A. S. Batsanov, J. A. K. Howard and T. B. Marder, *Chem. Eur. J.,* 2012, **18**, 5022-5035.

[3] N. C. Bruno, M. T. Tudge and S. L. Buchwald, *Chem. Sci.,* 2013, **4**, 916−920.

[4] *Gaussian 09, Revision A.*1. M. J. Frisch, G. W. Trucks, H. B. Schlegel, G. E. Scuseria, M. A. Robb, J. R. Cheeseman, G. Scalmani, V. Barone, B. Mennucci, G. A. Petersson, H. Nakatsuji, M. Caricato, X. Li, H. P. Hratchian, A. F. Izmaylov, J. Bloino, G. Zheng, J. L. Sonnenberg, M. Hada, M. Ehara, K. Toyota, R. Fukuda, J. Hasegawa, M. Ishida, T. Nakajima, Y. Honda, O. Kitao, H. Nakai, T. Vreven, J. A. Montgomery, Jr., J. E. Peralta, F. Ogliaro, M. Bearpark, J. J. Heyd, E. Brothers, K. N. Kudin, V. N. Staroverov, R. Kobayashi, J. Normand, K. Raghavachari, A. Rendell, J. C. Burant, S. S. Iyengar, J. Tomasi, M. Cossi, N. Rega, N. J. Millam, M. Klene, J. E. Knox, J. B. Cross, V. Bakken, C. Adamo, J. Jaramillo, R. Gomperts, R. E. Stratmann, O. Yazyev, A. J. Austin, R. Cammi, C. Pomelli, J. W. Ochterski, R. L. Martin, K. Morokuma, V. G. Zakrzewski, G. A. Voth, P. Salvador, J. J. Dannenberg, S. Dapprich, A. D. Daniels, Ö. Farkas, J. B. Foresman, J. V. Ortiz, J. Cioslowski, D. J. Fox, Gaussian, Inc., Wallingford CT, 2009.

[5] A. D. Becke, *J. Chem. Phys.,* 1993, **98**, 5648−5652.

[6] C. Lee, W. Yang and R. G. Parr, *Phys. Rev. B: Condens. Matter,* 1988, **37**, 785−789.

[7] T. Yanai, D. Tew and N. Handy, *Chem. Phys. Lett.,* 2004, **393**, 51−57.

[8] T. Lu and F. Chen, *J. Comput. Chem.,* 2012, **33**, 580−592.

[9] J. Yuan, Y. Yuan, X. Tian, Y. Liu and J. Sun, *J. Phys. Chem. C,* 2017, **121**, 8091−8108.

[10] X. Tang, L.-S. Cui, H.-C. Li, A. J. Gillett, F. Auras, Y.-K. Qu, C. Zhong, S. T. E. Jones, Z.-Q. Jiang, R. H. Friend and L.-S. Liao, *Nat. Mater.* 2020, **19**, 1332−1338.

[11] C. E. Colwell, T. W. Price, T. Stauch and R. Jasti, *Chem. Sci.,* 2020, **11**, 3923−3930.

[12] T. Mio, K. Ikemoto and H. Isobe, *Chem. Asian J.*, 2020, **15**, 1355−1359.

[13] E. F. Pettersen, T. D. Goddard, C. C. Huang, G. S. Couch, D. M. Greenblatt, E. C. Meng and T. E. Ferrin, *J. Comput. Chem.,* 2004, **25**, 1605−1612.

[14] W. Kabsch, *J. Appl. Cryst.,* 1993, **26**, 795−800.

[15] G. M. Sheldrick, *Acta Cryst. A,* 2015, **71**, 3−8.

[16] G. M. Sheldrick, *Acta Cryst. A,* 2008, **64**, 112–122.

[17] C. Kabuto, S. Akine, T. Nemoto and E. Kwon, *J. Cryst. Soc. Jpn.,* 2009, **51**, 218−224.
